# Biomolecule-mimetic nanomaterials for photothermal and photodynamic therapy of cancers: Bridging nanobiotechnology and biomedicine

**DOI:** 10.1186/s12951-022-01691-4

**Published:** 2022-11-16

**Authors:** Peng He, Guozheng Yang, Danzhu Zhu, Hao Kong, Yendry Regina Corrales-Ureña, Lucio Colombi Ciacchi, Gang Wei

**Affiliations:** 1 College of Chemistry and Chemical Engineering, Qingdao University, Qingdao, 266071 People’s Republic of China; 2 Hybrid Materials Interfaces Group, Faculty of Production Engineering, University of Bremen, 28359 Bremen, Germany

**Keywords:** Biomimetic synthesis, Nanobiotechnology, Photothermal therapy, Photodynamic therapy, Cancers

## Abstract

Nanomaterial-based phototherapy has become an important research direction for cancer therapy, but it still to face some obstacles, such as the toxic side effects and low target specificity. The biomimetic synthesis of nanomaterials using biomolecules is a potential strategy to improve photothermal therapy (PTT) and photodynamic therapy (PDT) techniques due to their endowed biocompatibility, degradability, low toxicity, and specific targeting. This review presents recent advances in the biomolecule-mimetic synthesis of functional nanomaterials for PTT and PDT of cancers. First, we introduce four biomimetic synthesis methods via some case studies and discuss the advantages of each method. Then, we introduce the synthesis of nanomaterials using some biomolecules such as DNA, RNA, protein, peptide, polydopamine, and others, and discuss in detail how to regulate the structure and functions of the obtained biomimetic nanomaterials. Finally, potential applications of biomimetic nanomaterials for both PTT and PDT of cancers are demonstrated and discussed. We believe that this work is valuable for readers to understand the mechanisms of biomimetic synthesis and nanomaterial-based phototherapy techniques, and will contribute to bridging nanotechnology and biomedicine to realize novel highly effective cancer therapies.

## Introduction

Cancer has always been a major threat to human health, and its morbidity and mortality are increasing year by year, which is the biggest obstacle to increasing the average life expectancy of human beings [1, 2]. New cancer patients diagnosed worldwide in 2020 were approximately 19.1 million, and more than 10 million cancer deaths were reported [3]. Practical clinical treatments, including physical surgery, radiation, and chemotherapy have been used to treat cancer [4–6]. However, these treatments exhibit side-effects. For instance, surgery and chemotherapy may bring physical pain to patients and sometimes the treated patients revealed higher recurrence rates. Meanwhile, radiation therapy and chemotherapy would kill both normal cells and cancer cells in the treatment process, and most importantly both techniques will affect the patients’ immune systems. In recent years, promising methods for cancer treatment have been developed. These emerging methods include photothermal therapy (PTT) [7], photodynamic therapy (PDT) [8], microwave hyperthermia, chemodynamic therapy (CDT), and others [9]. Compared with general nanomaterials, biomimetic nanomaterials present higher biocompatibility and biodegradability, enhanced targeting efficacy, and reduced side effects on normal cells, consequently decreasing the secondary effects and increasing the probability of recovery [10]. With the development of nanotechnology and improvement of optical techniques, phototherapy techniques such as PTT and PDT combined with nanomaterials have been explored as feasible methods for cancer treatment in the last 20 years [11, 12]. Phototherapy has advantages compared to traditional treatments such as the simplicity of the application protocols, the site-specific targeting of the treatment, and low side effects. Although both PTT and PDT have exhibited high therapy performance to tumors, their treatment mechanisms are different [13]. In the PTT process, nanomaterials with photothermal conversion properties (PTC) are injected into the tumor environment, and light with a desired wavelength is irradiated in the area where the PTC nanomaterials are located, resulting in conversion of light to heat. This increase in heat triggers the apoptosis of tumor cells [7]. In the PDT process, the photosensitizers produce reactive oxygen species (ROS) when irradiated, promoting the killing of tumor cells by ROS [14].

PDT and PTT nanomaterials with tailored structure and function are required to generate ROS or produce heat efficiently, respectively. Traditional synthesis methods of phototherapy nanomaterials include self-assembly [15], nanoprecipitation [16], hydrothermal reaction [17], chemical reduction [18], and others. Some nanomaterials created by these methods exhibit high performance for in-vitro tumor treatment; however, they also reveal a few shortcomings, such as low biocompatibility, potential toxicity, weak targeting binding, and are difficult to modify [19–21], which hinders the transfer to clinical trials.

Biomimetic synthesis is an advanced technique for preparing functional nanomaterials using biomolecules with tailored functional properties [22–24]. The materials synthesized by this method are called “biomimetic nanomaterials”. These materials have exhibited great potential for cancer treatment through PTT and PDT. For instance, Jiang et al*.* reported the biomimetic synthesis of gold nanorods (GNRs) promoted by erythrocyte membranes, which exhibited high PTT efficiency for treating pancreatic ductal adenocarcinoma cancer [25]. Jin et al*.* studied the production of phthalocyanine nanoparticles via self-assembly for PTT therapy, that demonstrated high efficacy both in vitro and in vivo [26]. Yu and co-workers reported the fabrication of biomimetic nanoreactors for the synergistic treatment of tumor metastasis by PDT and starvation therapy [27]. In these cases, the biomimetic nanomaterials exhibited several characteristics such as tailored structure and morphology, regulated functions and properties, higher biocompatibility than nanomaterials synthesized by other methods, and targeting ability to tumor cells.

Previous relevant reviews on synthesizing functional nanomaterials for PTT and PDF summarized the limitations of these therapies such as poor light penetration, low photodynamic efficiency, and poor targeting ability [27]. They also systematically summarized the synthesis methods and mechanisms of nanomaterial photosensitizers and carrier nanoparticles (NPs) [28]. Wei et al*.* summarized the development of traditional multifunctional photothermal nanomaterials such as Au NPs, Ag NPs, carbon, semiconductors, and organic molecules; reporting also about novel photothermal nanomaterials such as Au–Ag bimetallic NPs, graphdiyne, selenides [29]. Zhao et al*.* summarized the self-assembly strategies of supramolecular photothermal nanomaterials, the mechanisms of photothermal effects, and the design of polymer nanomaterials, proteins, and small molecule photothermal nanomaterials [30].

**Fig. 1:**
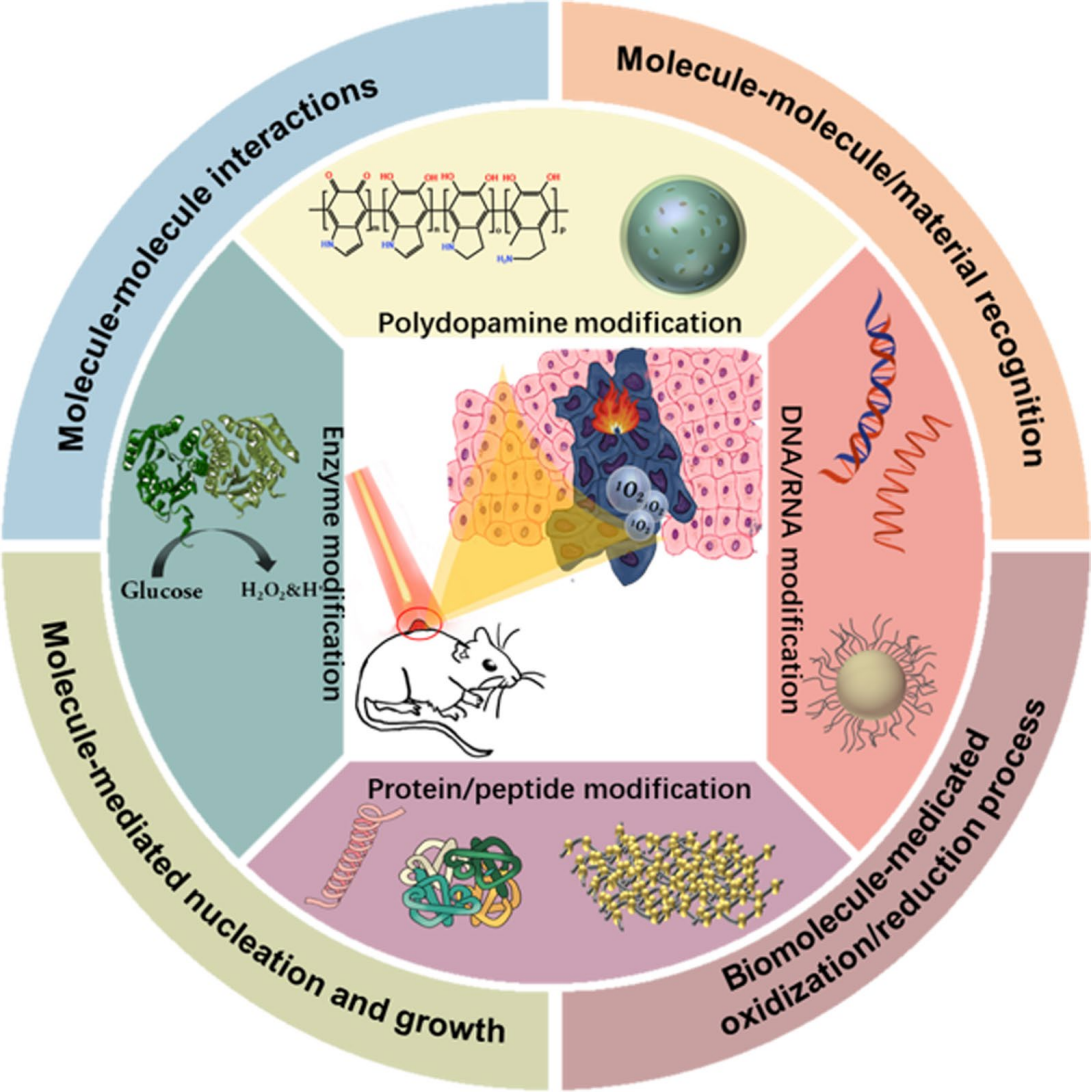
Schematic presentation on the synthesis, functional regulations, and PTT/PDT applications of biomolecule-mimetic nanomaterials

Considering the importance of biomolecule-mimetic nanomaterials in phototherapy, we aim to review the most recent advances in biomolecule-biomimetic nanomaterials (BBNM) for phototherapy, focusing on their use in biomedicine applications. However, we first introduce the general mechanisms of four synthesis methods used to produce nanomaterials and BBNM in wider application fields. These are shown schematically in Fig. 1.

## Biomimetic synthetic methods

In the process of biomimetic synthesis two or more raw materials are often used, so that there might be a complex interplay of interactions between the raw materials that leads to the nanomaterial self-assembly. Some easily accessible, “green” self-assembly driving forces, that are inherently environmentally friendly, are molecule–molecule interaction, biological recognition, molecule-inspired nucleation and growth, as well as redox reactions [31]. The self-assembly and synthesis process of nanomaterials can be tailored precisely using external stimuli, such as adjusting the pH, temperature, ionic strength, and other factors of the synthesis medium [32]. In addition, the bottom-up biomimetic synthesis of nanomaterials exhibits the advantages of more gentle reaction conditions, more tailorable structure and functions, and easier preparation than most top-down synthesis methods[23].

### Molecule–molecule interactions

**Fig. 2:**
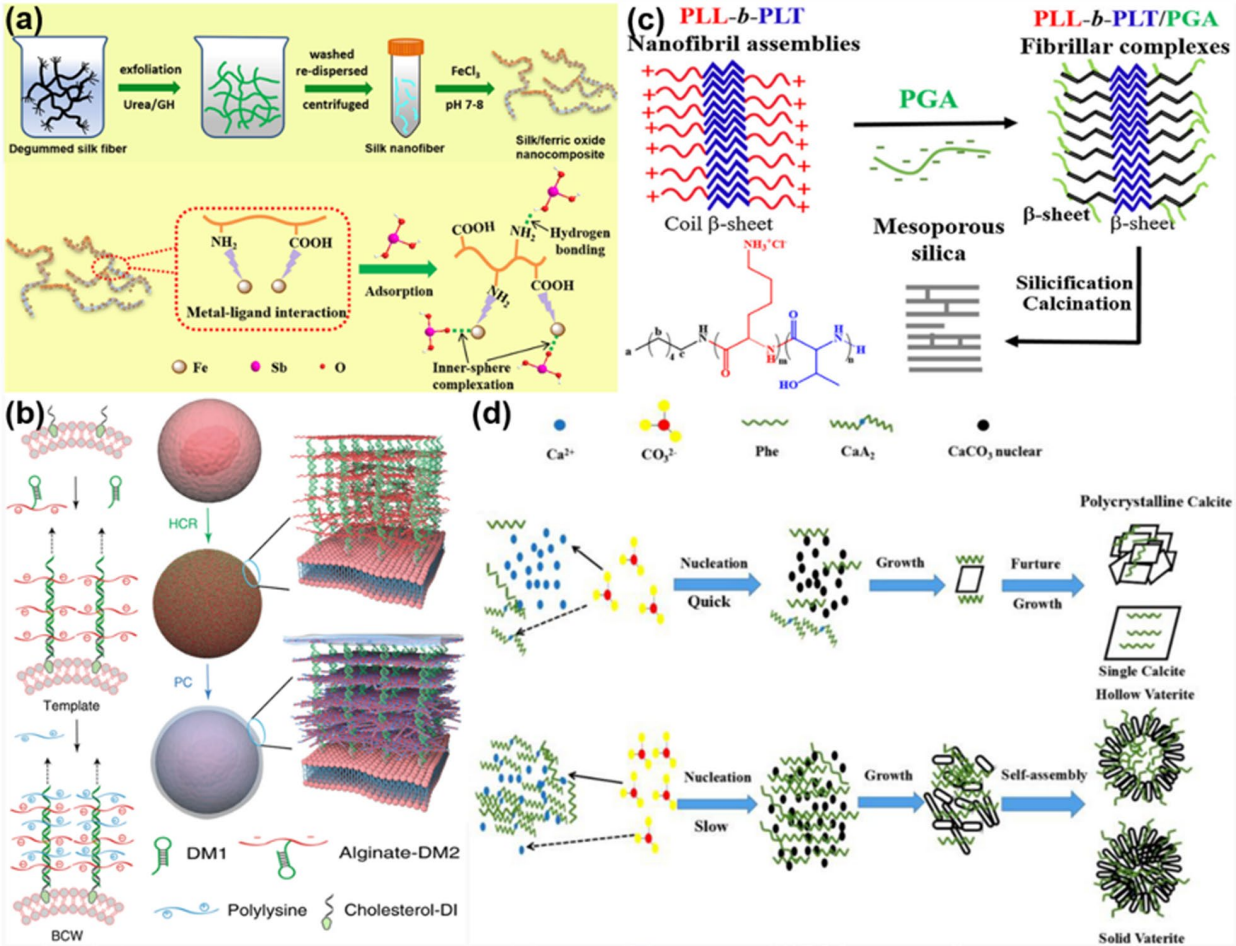
Biomimetic synthesis of nanomaterials based on molecule–molecule interactions: **a** in-situ synthesis of SF nanofiber-based Fe_2_O_3_ hybrid materials. Reprinted with permission from Ref. [33], Copyright 2021, Elsevier. **b** polypeptide-based self-assembly formation of BWCs for biosensors. Reprinted with permission from Ref. [36], Copyright 2016, Nature Publishing Group. **c** DNA hybridization mediated synthesis of mesoporous silica. Reprinted with permission from Ref. [35], Copyright 2016, Nature Publishing Group. **d** Amino acid -induced biomineralization of polycrystalline calcite. Reprinted with permission from Ref. [38], Copyright 2016, American Chemical Society

Molecule–molecule interactions can be divided into two classes: native intermolecular interactions and external interactions triggered by the outer stimulations of the molecules. Biomimetic synthesis can be achieved through hydrogen bonding, electrostatic binding, π-π interaction, hydrophobic segregation or their combination, such as in base pairing and stacking, or in ligand/receptor binding. It is a simple and fast synthesis method for synthesizing various inorganic and organic nanomaterials. Using the interactions between ligands and receptors, Qi and co-workers prepared silk fibroin (SF) nanofiber-supported Fe_2_O_3_ NPs for the removal of antimony [33]. As shown in Fig. 2a, SF nanofibers are firstly prepared by the hydrolyzation of SF by urea and guanidine hydrochloride, and then iron ions are added to the mixed solution, in order to synthesize SF nanofiber/Fe_2_O_3_ hybrid nanomaterials in-situ. The biomimetic hybrid nanomaterials are rich in amino and carboxyl groups, which can adsorb antimony-containing substances through hydrogen bonding. The enhanced removal efficiency towards antimony is attributed to the synergistic effects of both SF nanofibers and Fe_2_O_3_ NPs. Ye et al. demonstrated a one-pot biomimetic synthesis of hybrid organic–inorganic nanomaterials via the coordination between metal ions in posnjakite (Cu_4_(SO_4_) (OH)_6_·H_2_O) and amino groups of streptavidin. A novel hybrid nanomaterial with enhanced ability for immunoassay labeling was synthesized combining a signal-amplification enzyme (horseradish peroxidase, HPR), and copper ions (Cu^2+^) by co-precipitation[34].

In another study, Shi and co-workers prepared biomimetic cell walls through the design of self-assembled DNA templates and biomacromolecules [35]. As indicated in Fig. 2c, alginate and DNA were chemically combined to form alginate-DNA monomers (Alginate-DM2), which can form a hairpin structure with complementary DNA sequences (DM1) through DNA hybridization. The mammalian cell membrane was self-coated by means of a hybridization chain reaction (HCR). A polyelectrolyte complex between polylysine and the hairpin coated the cell surface. Chen et al. produced block polypeptide protofibril complexes through hydrogen bonding, which could be used as both nucleating agents and templates to synthesize polypeptide/SiO_2_ NPs and films [36]. As shown in Fig. 2b, PLL and poly-threonine (PLT) self-assembled through hydrogen bonding to form sheet-like PLL-*b*-PLT nanomaterials. In addition, PLL-*b*-PLT bound to poly-glutamic acid (PGA) through electrostatic interactions to form PLL-*b*-PLT/PGA polypeptide composite nanofibers, in which the hydroxyl groups of the threonine residues promoted the nucleation and biosynthesis of SiO_2_ NPs.

Besides intramolecular interactions, temperature, pH, light, electric fields, and other external stimuli may affect intermolecular recognition and binding. The modulation of intermolecular recognition can be achieved by changing the external conditions, or bestowing the designed materials with a specific function after adjusting the experimental conditions. For instance, Tofanello et al*.* designed a basic polypeptide sequence AAAXCX, in which X is an amino acid containing lysine or arginine residues. They were able to achieve different degrees of reduction of gold nanoparticles (AuNPs) with the designed peptides by changing the pH of the reaction [37]. Yang et al. tailored the biomineralization of crystalline calcium carbonate (CaCO_3_) by controlling the concentration of phenylalanine (Phe) and the flow rate of CO_2,_ as shown in Fig. 2d. During the biomineralization process, when the concentration of Phe in the solution was low, the concentration of Ca^2+^ high, and there was a flow of CO_2_, Ca^2+^ and CO_3_^2−^ ions rapidly nucleated and grew into large CaCO_3_ particles. On the other hand, when the concentration of Phe in the solution was high and the concentration of Ca^2+^ low, the nucleation rate of small-sized CaCO_3_ particles was favored, forming nanoflowers due to the high coverage with Phe. [38]. In another case, Li and co-workers achieved fine tuning of hydroxyapatite (HAp) by controlling the system temperature, enabling the synthesis of HAp NPs and nanorods [39]. Their study indicated that HAp nanorods with an aspect ratio of 2.0–4.4 and good biocompatibility could be biomimetically synthesized by adjusting the reaction temperature at 30 °C.

### Molecule–molecule/material recognition

The specific recognition between molecules has the advantages of strong binding ability and high specificity to recognize the corresponding structure or materials in a complex environment. Based on the characteristics and ability of specific recognition between antibodies and antigens, targeting functions of cells, and others, many biomolecule-based composite materials have been created by simulating the recognition function between molecules. These recognition properties of the nanomaterials allow for the targeting of specific areas where PDT and PTT could be applied in the future.

**Fig. 3:**
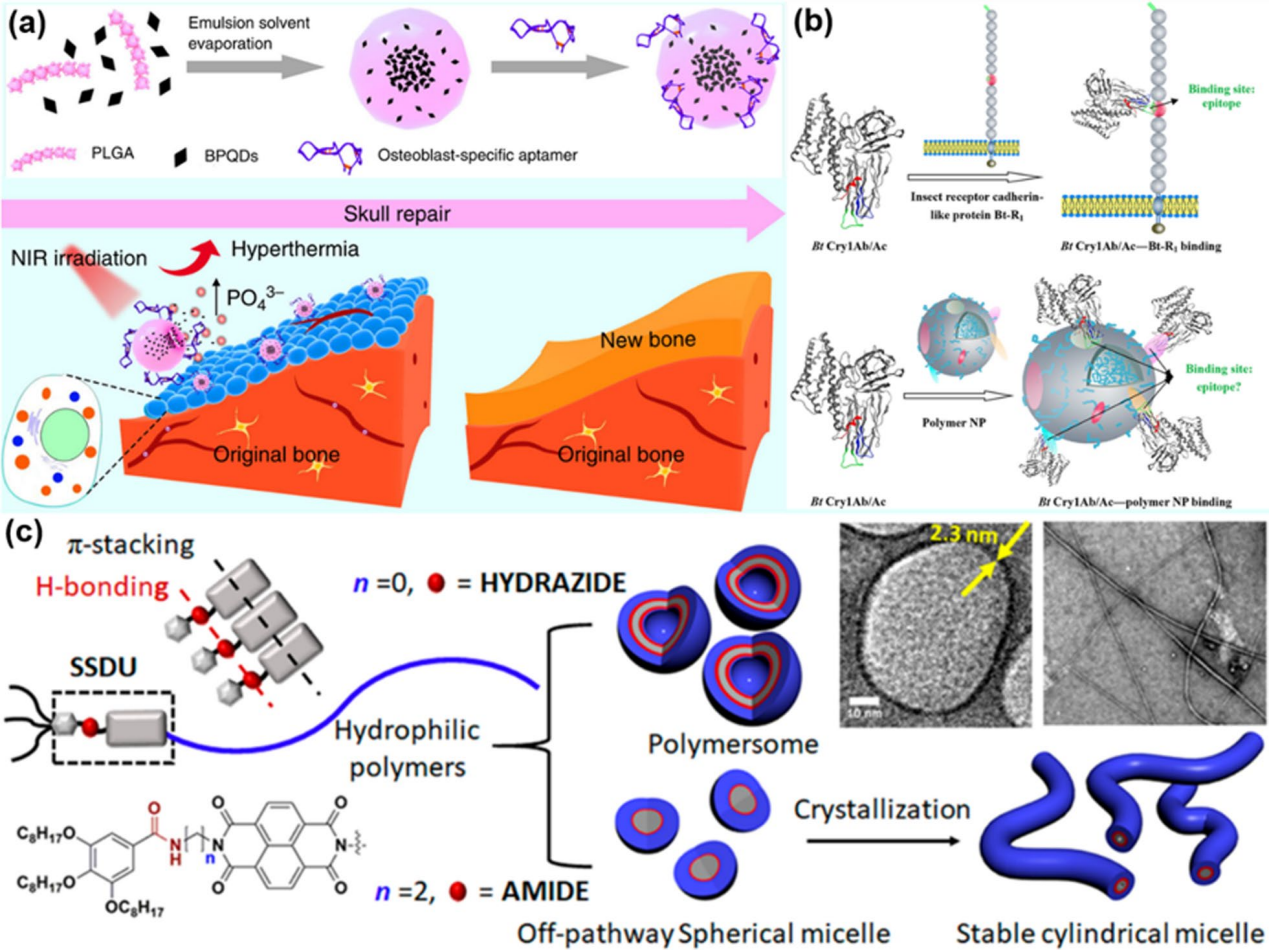
Molecule–molecule/material recognition promotes the formation of bionanomaterials: **a** polymer-encapsulated BPQDs for the synthesis of bioinspired MVs for bone tissue engineering. Reprinted with permission from Ref. [40], Copyright 2019, Nature Publishing Group. **b** biomimetic polymer NPs with specific binding ability with Bt Cry1Ab/Ac. Reprinted with permission from Ref. [41], Copyright 2018, American Chemical Society. **c** design and functionalization of SSDU for biomimetic supramolecular cylindrical micelles. Reprinted with permission from Ref. [42], Copyright 2021, American Chemical Society

Biological tissues contain both extracellular vesicles (EVs) and matrix vesicles (MVs). MVs can transmit cellular information and regulate cellular mineralization. Wang et al. reported the preparation of bioinspired MVs by modifying black-phosphorus quantum dots (BPQDs) encapsulated within poly (lactic acid-glycolic acid) NPs (PLGA NPs) with aptamers (Apt), which can guide the biomineralization of calcium phosphate to form bone materials [40]. As presented in Fig. 3a, BPQDs were firstly encapsulated in the PLGA NPs, and, then the PLGA NPs surface was modified with osteoblast-targeted nucleic acid Apt to obtain bioinspired MVs with targeting and mineralization abilities. It was found that the designed Apt-MVs can enter the body, reach osteoblasts under the targeting guidance of Apt, and then be degraded to be the source of phosphates for new bone biomineralization. In a similar study, Liu and co-workers synthesized abiotic polymer NPs by mimicking the mutual recognition between proteins in microorganisms [41]. As indicated in Fig. 3b, Bacillus thuringiensis (Bt Cry1Ab/Ac) protein ligands have high affinity for cadherin-like Bt-R1 receptors. This was exploited to synthesize polymer NPs with specific binding ability to three recognition sites of Bt Cry1Ab/Ac. The polymer NPs exhibited low recognition towards other Bt proteins, and therefore could inhibit the effects of other proteins, achieving high-performance separation and extraction of Bt Cry1Ab/Ac.

Molecular recognition can promote the self-assembly of various molecules and the formation of biomimetic supramolecular nanomaterials. For instance, surfactants or amphiphilic block polymers can usually form different nanostructures based on different hydrophilic and hydrophobic groups at the two molecular ends. In previous studies, the Ghosh group has reported the design of a hydrophobic supramolecular-structure-directing-unit (SSDU) with self-recognition function, which could guide molecular self-assembly to form different nanostructures by tailoring the functionalization of SSDU with various groups [42]. Figure 3c shows the design principle of SSDU containing amide groups. It was found that the self-assembly and recognition of designed SSDU induced the formation of biomimetic supramolecular cylindrical micelles.

### Molecule-mediated nucleation and growth

To nucleate and growth bionanomaterials, some factors such as intermolecular interactions, self-assembly, and molecule-nucleation agent interactions are crucial. Molecular-mediated nucleation usually neither require extra materials to provide sites for nucleating substances, nor require additional reducing agents. Besides, the use of molecular templates to mediate the nucleation and growth of hybrid materials can achieve one-step reduction and polymerization with high synthesis efficiency. Usually, molecule-mediated nucleation and growth of nanomaterials can achieve fine control of the morphology and structure of nucleated crystals by controlling the growth time and ionic concentration. More importantly, small molecules can be added during the nucleation process to improve the properties of bionanomaterials, including their antibacterial property, adsorption, separation, biocompatibility, enzymatic activity, and others.

**Fig. 4:**
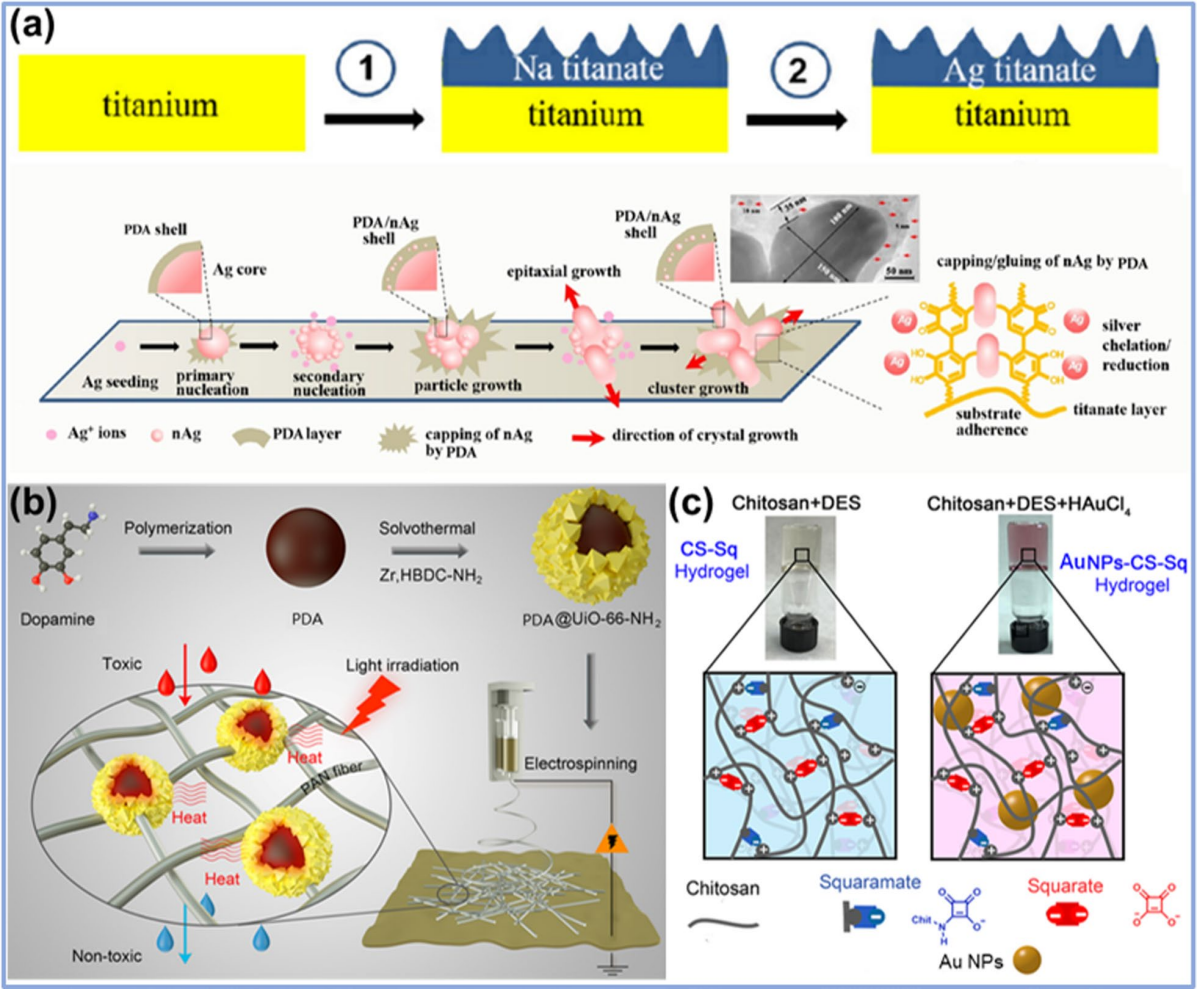
Biomimetic synthesis of nanomaterials via nucleation and growth process: **a** PDA-mediated synthesis of nAg/PDA core/shell hybrid nanoparticles. Reprinted with permission from Ref. [44], copyright 2021, Elsevier. **b** PDA-mediated synthesis of PDA@UiO-66-NH_2_ nanoparticles. Reprinted with permission from Ref. [45], Copyright 2019, American Chemical Society. **c** DES-mediated synthesis of AuNPs in CS-Sq hybrid hydrogels. Reprinted with permission from Ref. [46], Copyright 2020, American Chemical Society

For instance, Qiu and co-workers designed and synthesized metal–organic framework (MOF) crystals with controllable size and morphology, employing the molecule-mediated nucleation method [43]. In the synthesis process, short-chain starch nanoparticles were used as seeds for the growth of cyclodextrin-based MOF (CD-MOF) with good degree of crystallinity, by which CD-MOF crystals with adjustable size could be synthesized by controlling the kinetics of nucleation and growth. Meanwhile, it was possible to encapsulate a natural derivative of polyphenol, resveratrol, into the CD-MOF crystals to form functional biomimetic nanomaterials, which could significantly improve the stability of resveratrol and enhance their anticancer and antibacterial performances. Jia et al. synthesized a nanosilver (nAg)/polydopamine (PDA) core/shell hybrid material (nAg/PDA) using a biomolecule-mediated nucleation strategy [44]. As shown in Fig. 4a, sodium titanate was first grown on a titanium template by a hydrothermal method. After the ion exchange between Ag^+^ and Na^+^, silver titanate was formed on the titanium template. Then, dopamine (DA) was added to the solution to reduce adsorbed Ag^+^ and mediate in-situ nucleation and growth of nAg, and meanwhile form PDA through the self-polymerization on the surface of nAg core. It was found that the morphology of nAg/PDA materials can be adjusted into spherical particles, grape-like clumps, and dendritic clusters by controlling the reaction time. In another similar study, Yao et al. synthesized UiO-66-NH_2_-capped PDA NPs using the chelation reaction between the catechol groups in DA with metals in MOF. The heterogeneous nucleation of UiO-66-NH_2_ on the surface of PDA NPs promoted the formation of PDA@ UiO-66-NH_2_ core/shell NPs (Fig. 4b) [45].

Castellanos et al. used chitosan (CS) hydrogels as 3D scaffolds to provide nucleation sites for the biosynthesis of AuNPs to form plasmonic hybrid nanomaterials [46]. As presented in Fig. 4c, CS, diethyl squarate (DES), and HAuCl_4_ were mixed to form CS-squaric acid (CS-Sq) hydrogels, in which DES serves as the reducing agent of HAuCl_4_ to medicate the biomimetic synthesis of AuNPs. Meanwhile, the 3D hydrogel presents nucleation and growth sites for stabilizing the formed AuNPs, leading to AuNPs distributed uniformly in the fabricated CS-Sq hydrogels. In this biomimetic process, the AuNPs could be regulated by adjusting the concentrations of CS and HAuCl_4_.

### Molecularly mediated oxidation/reduction

Amino acids, as the monomers of proteins, play indispensable and important roles in organisms and also serve as excellent templates for the biosynthesis of functional nanomaterials. The strategy of using amino acids as ligands to synthesize and encapsulate metal nanoparticles has been widely utilized for the biomimetic synthesis of various materials.

**Fig. 5:**
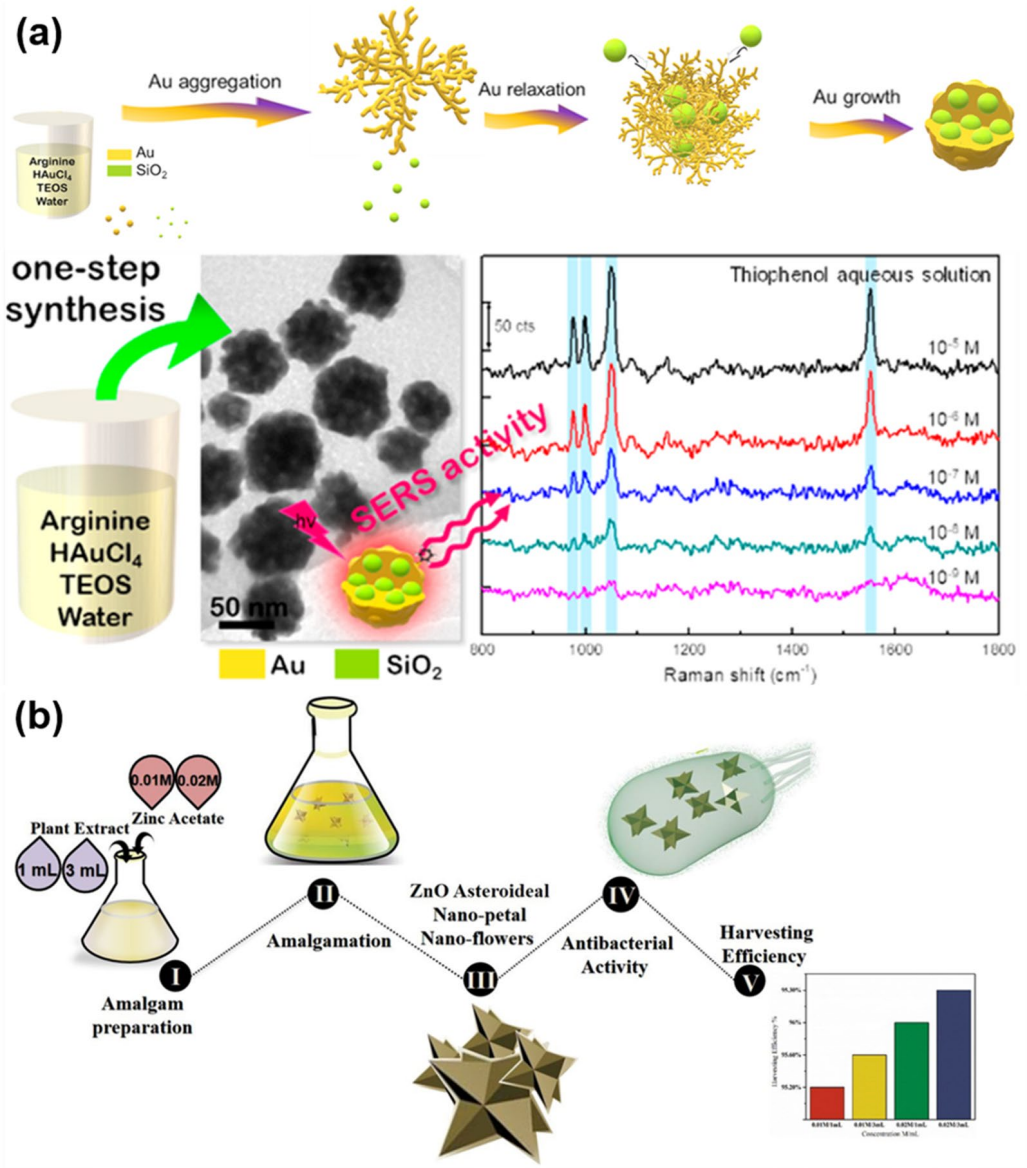
Biomolecule-induced reduction for biomimetic synthesis of nanomaterials: **a** AA-induced reduction of gold salt to AuNPs with SERS activity. Reprinted with permission from Ref. [48], Copyright 2020, American Chemical Society. **b** Plant extract-induced formation of ZnO NFs with antibacterial activity and photochemical properties. Reprinted with permission from Ref. [49], Copyright 2021, Elsevier

Li and co-workers synthesized biomimetic AuNPs using amino acid-dithiocarbamate (AA-DTC) as a ligand to induce the reduction of gold salt to metallic NPs [47]. Glycine (Gly), glutamic acid (Glu), and histidine (His), differing in their isoelectric point, were selected as neutral, acidic, and basic amino acids for the synthesis of AA-DTCs through the reactions with CS_2_. In the synthesis process, pyrolysis of the AA-DTCs promoted the reduction of HAuCl_4_ into AuNPs. Meanwhile, DTC acted as a monoanionic ligand to connect with the surface of AuNPs to form DTC-encapsulated AuNPs. The biomimetic AuNPs exhibited high surface-enhanced Raman scattering (SERS) activity and revealed visible peak intensity at low concentration. In addition, the formed AuNPs presented low toxicity and good biocompatibility; therefore, they could be utilized as the nanoprobes of SERS for in-situ cell imaging. In another similar study, Zhou et al. employed the AA-mediated reduction strategy to synthesize AuNPs using the basic amino acid (arginine) as both reducing and encapsulating agent for AuNPs [48]. Figure 5a shows the biomimetic synthesis process, in which a one-step hydrothermal reaction is utilized to create pomegranate-like SiO_2_@Au hybrid nanomaterials. Arginine acts as a catalyst to catalyze the formation of SiO_2_, and promotes the combination of AuNPs and SiO_2_, thereby realizing the formation of SiO_2_@Au hybrids. The SiO_2_@Au nanohybrids had strong SERS activity and could be used to prepare paper-based SERS-active substrates. The SERS-based detection limit was about one ng towards thiophenol.

In addition to AA, some natural product extracts can also be used as reducing agents for the biomimetic synthesis of metallic NPs. Compared to chemical reducing agents such as NaBH_4_ and citrate acid, phytochemical manufacturing methods of metallic NPs are more eco-friendly, and have attracted great attention in recent years. In a typical case, Hasan and co-workers reported the biomimetic synthesis of ZnO nanoflowers (ZnO NFs) using the extract of Withania coagulans as reducing and encapsulating agent and zinc acetate as precursor [49], as shown in Fig. 5b. The as-synthesized ZnO NFs exhibited large specific surface area, good antibacterial properties, and fine-tuning of their morphology. Through the same biomimetic synthesis method, Pan et al*.* used astragalus extract as a reducing agent and encapsulant for the biomimetic synthesis of ZnO NFs, which effectively avoided the aggregation of ZnO NFs during the synthesis process [50]. The biomimetic ZnO NFs synthesized from astragalus extracts revealed good antibacterial, antioxidant, and electrochemical properties, and the detection limit of the fabricated electrochemical sensor (ZnO NF-modified electrode) towards 4-nitrophenol reached 0.08 μM, which was mainly attributed to enhanced electron transfer in the detection process caused by the phenolic compounds in the astragalus extract. In a similar study, Chinnappan et al*.* used Bauhinia extract as a reducing agent for the green synthesis of silver nanoparticles (AgNPs), and the biomimetic AgNPs exhibited good antibacterial properties [51]. Dadashpour et al*.* reduced Ag^+^ into AgNPs with chamomile extract through a biomimetic synthesis method [22]. In the synthesis process, the AgNPs were encapsulated by the chamomile extract. After combining biomimetic AgNPs with chamomile, the finally synthesized AgNPs revealed good dispersibility in a short time and anti-cancer properties [52].

## Biomolecular functional nanomaterials

Biomolecules have unique structural and functional advantages, and they have a wide range of applications in the field of biomedicine and in photothermal and photodynamic therapy. In this section, we introduce the functions and biomedical applications of common biomolecular proteins, peptides, DNA, RNA, PDA, and enzymes.

### DNA and RNA-based nanomaterials

DNA is a biomolecule composed of nucleotides, with a sequence that can be designed and accurately recognized by organisms. Recently, much literature on DNA-based synthesis., self- formation, and structure design strategies of nanomaterials [31, 53], including 0D NPs, 1D nanowires/nanotubes, 2D arrays/films, and 3D hydrogels have been reported.

**Fig. 6:**
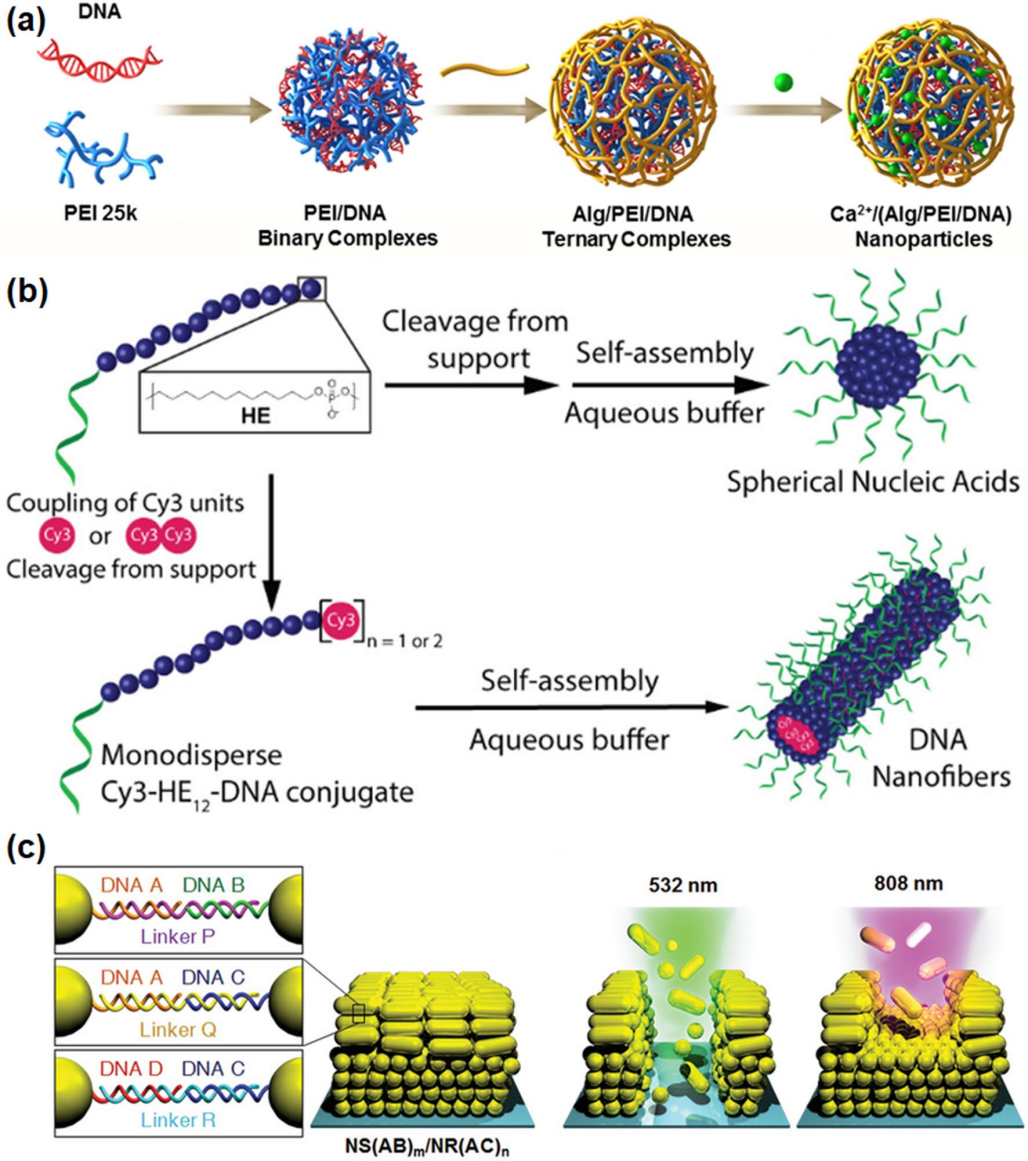
**a** Schematic illustration of the preparation of biomimetic DNA/polymer NPs. Reprinted with permission from Ref. [57], Copyright 2018, Elsevier. **b** Sequence-controlled synthesis of dye/DNA conjugates and their self-assembly behavior. Reprinted with permission from Ref. [60], Copyright 2018, American Chemical Society. **c** Illustration of the multi-domain NP film composed of nanospheres and nanorods. Reprinted with permission from Ref. [64], Copyright 2022, Wiley–VCH

Using DNA nanotechnology, Vries et al. replaced the hydrophobic polymer unit of a DNA block copolymer delivery system with several alkyl chain-modified 2-deoxyuridine nucleotides (U) [54]. DNA amphiphilic molecules can self-assemble into micellar NPs by microphase separation in an aqueous environment. In the absence of any targeting units, these NPs can adhere to corneal tissue for a long time[55, 56]. In addition, they modified and extended the complementary ssDNA to enable it to bind drug molecules as functional drug carriers. In another case, Zhang et al. chose sodium alginate (Alg) as the shielding material, introduced Ca^2+^ as a cross-linking agent to cross-link the carboxyl groups onto Alg, and successfully developed Ca^2+^/(Alg/PEI/DNA) NPs [57]. The formed biomimetic NPs were shown to have excellent stability to proteins and particles, could improve the transfection efficiency, enhance the cellular uptake, and reduce cytotoxicity, and therefore can be used as an effective nano-transfer system for both gene delivery and tumor therapy (Fig. 6a).

Rolling circle amplification (RCA) technology is an interesting technique for preparing DNA NPs, which can rely on the strong strand displacement properties and high processivity of DNA polymerase to make repeated copies of templates and assemble long ssDNA products. The downside of this technique is the inability to control the size of the resulting DNA NPs. To address this issue, Lee et al*.* reported an RCA-based DNA NP synthesis strategy that controls the size of the RCA product by embedding secondary-structure-forming sequences and positive-modifying nucleotides in the replication template. Complementary sequences and chemical modifications of nucleobases enabled the synthesis of DNA NPs with desired nanoscale size [58]. In a similar study, Tran et al*.* used RCA technology to self-assemble and synthesize DNA NPs and incorporated them into aptamer-drug conjugates, in order to improve the in vivo stability and targeting specificity of biomimetic DNA nanomaterials [59].

Supramolecular self-assembled 1D structures have attracted great attention for drug delivery and templating of complex linear arrays due to their high aspect ratio and rigidity. In general, the assembled structure of amphiphiles and polymers can be controlled by changing the relative bulk amounts of the hydrophilic and hydrophobic moieties; however, as reported by Bousmail et al*.* [60], the introduction of a single cyan (Cy3) dye molecule at a specific site of the DNA sequence could completely transform the overall structure into 1D DNA nanofibers in aqueous medium (Fig. 6b). The length of the DNA nanofibers can be controlled by adjusting the concentration of added monomers. High aspect ratio fibrous structures have shown longer blood circulation time and higher cellular uptake than spherical particles, and could be used as a target for drug delivery and biosensing.

Inspired by a non-viral gene delivery system (GDS), Zhang et al. successfully designed and synthesized a small-molecule gene vector (TR4) with aggregation-induced emission (AIE) properties by exploiting the self-assembly of pDNA [61], which has a similar function as cell-penetrating peptides. The designed TR4 could be used as a novel GDS, in which the positively charged region is a short peptide consisting of four arginine residues modified with two hydrophobic moieties. This structure can not only increase the transfection efficiency, but also effectively reduce the risk of increasing cytotoxicity elicited by arginine residues.

Due to the programmable molecular design and controllable self-assembly, DNA has been utilized as an excellent precursor for the fabrication of biomimetic materials with complex functions [53, 62, 63]. For instance, Kim et al*.* reported an assembly strategy to construct 2D multicomponent NP films by combining a “bottom-up” DNA-directed layer-by-layer (LbL) assembly strategy with a “top-down” plasmonic photothermal patterning (Fig. 6c). The NP film was made up of stacks of domains, each composed of particles with different plasmonic properties and grafted with a different combination of DNA sequences. Compared with the traditional lithography technique of sequential etching, this strategy can control the laser to change the selective removal of target regions in the film, so that the selected DNA sequence carrying information can shrink and expand in a programmable way, resulting in highly oriented structural deformation [64]. In another case, Mitta et al. prepared AuNPs by drop-casting and embedded them into DNA films to obtain composite films with high stability and enhanced response to UV light, indicating that 2D DNA films can be used as carriers for the modification and functionalization of biomimetic nanomaterials [65].

3D nano-hydrogel materials assembled from DNA present a responsive behavior to various stimuli, and can serve as biosensing platforms [66]. For example, Pandey et al*.* synthesized biomimetic carbon dots (CDs) using DNA as a precursor and further used them as cross-linking agents to prepare DNA-based hybrid fluorescent hydrogels with interconnected networks and a hierarchical porous structure [67]. The formed DNA-biomimetic CDs revealed very low cytotoxicity, and the formed hybrid hydrogel could be effectively internalized into microbial cells. In addition, this composite hydrogel exhibited selective detection behavior towards dopamine, revealing potential application as a good biosensing platform.

As a natural biopolymer, RNA has different sequences, secondary structures, and tertiary and quaternary interactions than DNA [68]. Unlike DNA, RNA is more flexible in structure and more versatile in function [69]. Over the past decades, RNA nanotechnology has grown exponentially. RNA NPs can be designed in different sizes and shapes while maintaining high thermal stability for in vivo applications [70]. Bui and co-workers reported the design and construction of fine-tuned triangular RNA nanostructures with a four-U-helix linking motif [71]. The highly versatile properties of complex RNA nanomaterials have also been demonstrated by fabricating equilateral RNA/DNA polygons such as rectangles, pentagons, and hexagons, which exhibited great potential to be expanded into larger polygonal structures. In another study, Jasinski et al*.* controlled the size of RNA polygons by adjusting the number of base pairs between each vertex, and constructed RNA triangles, squares, and pentagons [72]. They further explored the effect of the size and shape of RNA NPs on the biofunctions, and indicated that the circulation time of RNA NPs was related to their size, and the elimination pathway was related to their shape. This study contributes to the use of the unique and easily tunable properties of RNA NPs for nucleic acid nanotechnology and drug/gene delivery.

### Peptide and protein-based nanomaterials

The self-assembly and aggregation of peptides and proteins play a key role in many human body functions. Peptides are short amino acid sequences that can act as structural and functional supports for naturally occurring proteins, and can therefore be used as biomimetic building blocks for supramolecular self-assembly. [73–75]. Amyloid aggregates are ordered β-sheet-rich supramolecular structures assembled from a variety of peptides and proteins. Over the years, the association between amyloid structures and disease states has been discovered, and the mechanisms of toxicity of various amyloid species ranging from oligomers to mature amyloid nanofibrils have been understood through numerous studies [76–78]. Currently, more attention has been paid to using amyloid fibrils as templates or building blocks to design biomimetic nanomaterials with different structures for biomedicine and tissue-engineering applications.

**Fig. 7:**
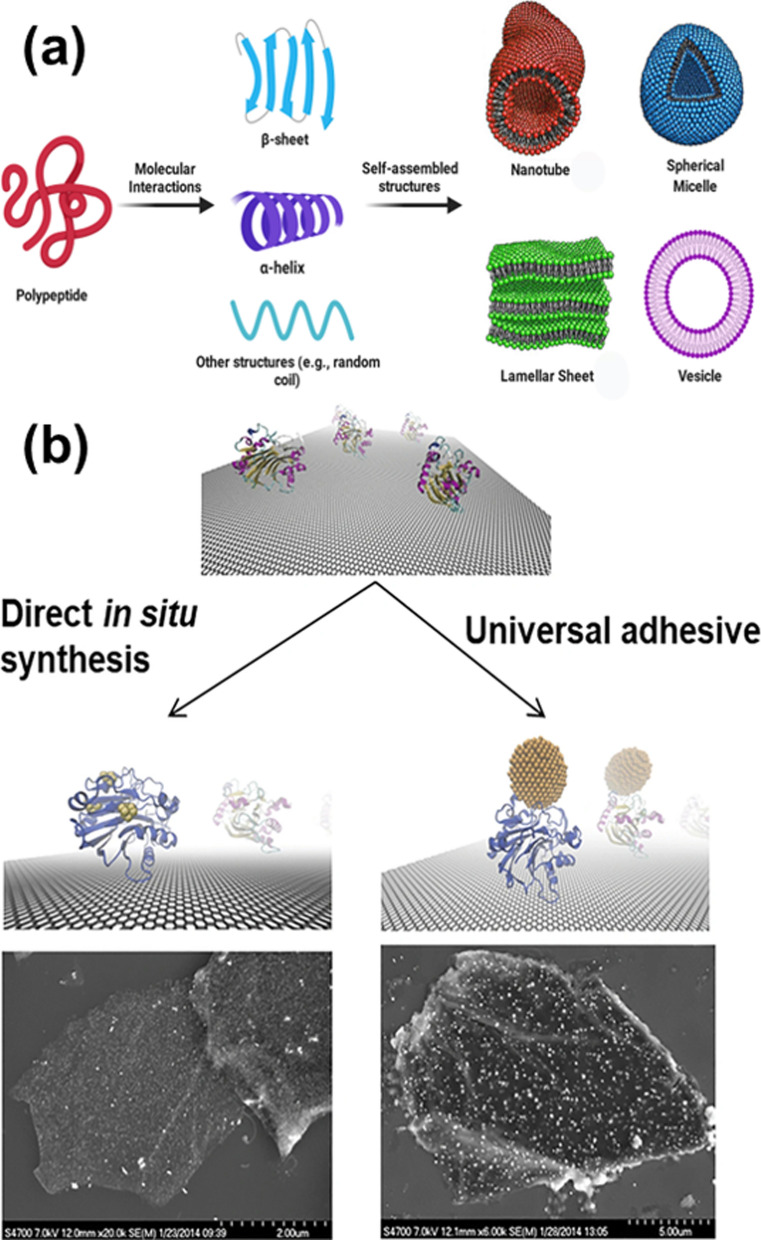
**a** Self-assembly of amphiphilic peptides into various nanostructures. Reprinted with permission from Ref. [79], Copyright 2021, Elsevier. **b** protein-mediated biomimetic synthesis of AuNCs on rGO. Reprinted with permission from Ref. [89], Copyright 2020, Springer

Supramolecular peptide self-assembly has great flexibility and versatility for the design and construction of various advanced nanomaterials with specific functions, and can form various nanostructures [79] (Fig. 7a). The self-assembly process of peptides is generally driven and controlled by non-covalent interactions between molecules, such as hydrogen bonding, electrostatics, hydrophobicity, van der Waals forces, and π-π interactions. Peptide-based nanomaterials in the form of fibrils can be used for many biotechnological applications, including tissue engineering [80], immunotherapy of infectious diseases or cancer [81], and drug delivery [82].

The longitudinal assembly of peptide nanofibers is generally difficult, and the length of the nanofibers is closely related to their application, such as in hydrogels. How to rationally design the peptide nanofibers with a fixed length is crucial for their applications. The length of peptide nanofibers can be effectively controlled by rationally designing the structure of polypeptides [83]. Peptide-based hydrogels have good biocompatibility and degradability, and they have wide applications in drug delivery and immune vaccines. For instance, Han et al*.* added a cardioprotective peptide (GHRPS) and a peptide sequence (GTAGLIGQ) that can be degraded by matrix metalloproteinase 2 (MMP-2) to a novel peptide amphiphile (PA), which could self-assemble into peptide hydrogels in the presence of NapFF [84]. The prepared peptide hydrogels can encapsulate exosomes for improving cardiac function after myocardial infarction. In addition, the targeting of specific peptide molecules can also be used for the delivery of certain nano-drugs. Combining specific targeting peptides with chemotherapeutic drugs or nanotherapeutic platforms can greatly enhance their therapeutic efficacy.

Vrettos et al*.* summarized a variety of functional peptides that can target specific overexpressed proteins in cancer cells, such as RGD, GnRH, and EGF [85]. Cai et al*.* used the novel F3 peptide in combination with maleimide-polyethylene glycol-polylactic acid conjugate (mal-PEG-PLA) to obtain tumor-cell-targeting nanotherapeutics [86]. The use of a cell-targeting peptide improved the anti-tumor effect of the drug system significantly. Their work provides a new design idea for using the targeting function of peptide molecules to improve the utilization of drugs.

Specific nanostructures can be obtained by adjusting the amino acid sequence and some physicochemical properties to affect the self-assembly of proteins to form different nanomaterials, including nanofibers, nanospheres, nanotubes, nanosheets, and others [79]. Exploring specific conditions to obtain ideal protein nanostructures is of great significance for the application of proteins in biomedicine. Lu et al. designed silk fibroin and zein (a hydrophobic plant protein) by an assembly method to prepare nanosphere drug carriers with controllable particle size. Two antitumor drugs, paclitaxel (PTX) and curcumin (CUR), were loaded into the nanosphere carriers, which could effectively improve the sustained-release performance of the drug and reduce the damage to normal cells [87]. In addition, the rich binding sites of proteins and a variety of reactive groups can be used to prepare functional nanomaterials [88]. For instance, Griep et al*.* used bovine serum albumin (BSA) to reduce graphene oxide (GO) to obtain biologically-active BSA-rGO composites, and further synthesized BSA-rGO-supported gold nanoclusters (AuNC) through an in-situ reduction method [89] (Fig. 7b). The created biomimetic hybrid nanomaterials could serve as a multifunctional nanoplatform for the activity and concentration detection of trypsin.

### PDA-based nanomaterials

PDA is a substance with a wide range of applications in the field of materials science and biomedicine. Due to its high biocompatibility, good biodegradability, easy modification, and feasible functionalization, and certain photothermal conversion capabilities, it has been widely used in drug delivery, cancer treatment, medical imaging, metal nanomaterial modification, and others [90]. The research of PDA originated from the mussel adhesion protein, which has a large number of catechol and amino groups and thus reveals strong adhesion properties. PDA can be obtained by the oxidation of catecholamines such as dopamine, which is an excellent biological coating and can be used to cover various nanomaterials by simply increasing the solution pH and then undergo further modification to obtain multifunctional biomimetic nanomaterials.

**Fig. 8:**
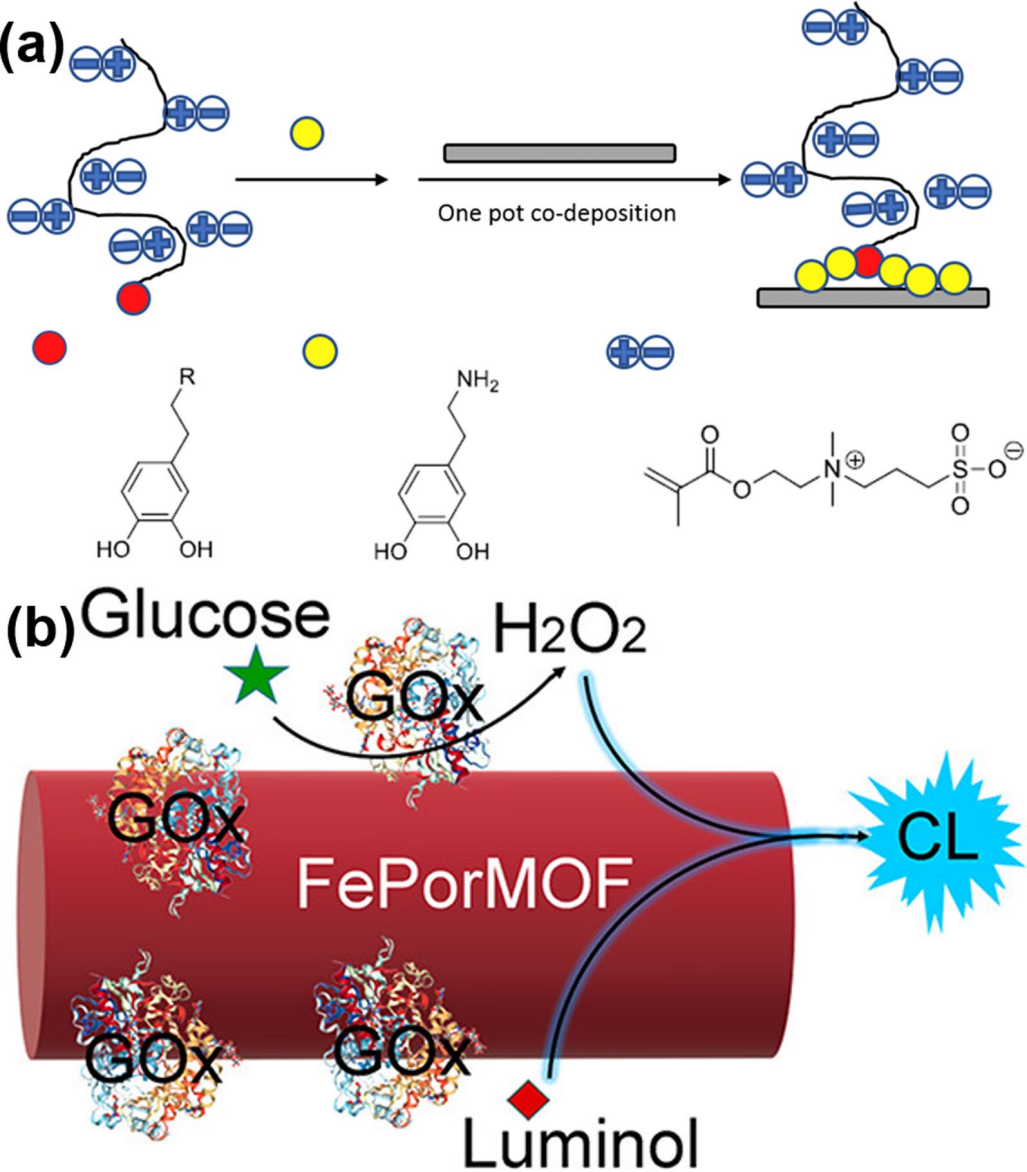
**a** Process of co-deposition of PDA and PSB. Reprinted with permission from Ref. [91], Copyright 2019, Elsevier. **b** Principle of FePorMOF/GOx CL system. Reprinted with permission from Ref. [97], Copyright 2019, American Chemical Society

Asiyeh et al*.* designed a neural probe based on PDA and a zwitterionic material (poly sulfobetaine methacrylate, PSB) through co-precipitation [91] (Fig. 8a). The use of PDA-PSB as a coating reduces the adverse reactions of electrodes implanted in biological tissues, and greatly improves the biocompatibility and stability of neural probes. It was found that PDA can be prepared into various nanostructures, such as NPs and nanocapsules [92]. The particle size of PDA NPs is an important factor affecting its functions, and the conditions such as pH, concentration, and temperature are main factors that affect the size of PDA NPs. Huang et al*.* investigated the use of boric acid and Lewis base to adjust the particle size of PDA NPs [93]. The properties of the prepared PDA NPs with different particle sizes were explored, which provided a guiding method for the synthesis of PDA NPs.

At the same time, PDA is a kind of material with high affinity for a variety of metal ions and metal NPs, which provide high potential for the creation of biomimetic nanomaterials. The large amount of catechol on the surface of PDA provides a strong interaction and redox effect with metal ions, acting as a very suitable metal material carrier [94]. Li et al*.* explored the use of PDA as a carrier to support Pt^4+^ and its reduction to Pt NPs in aqueous solution at room temperature without the addition of other reducing agents [95]. The reduced Pt NPs (about 20 nm in diameter) could be uniformly dispersed on the PDA film and exhibited excellent catalytic activity. This study provides a simple biomimetic synthesis method for the preparation of PDA/metal NP composites.

### Enzyme-based nanomaterials

Enzymes play a key role in living organisms because they participate in complex physiological and catalytical processes. They could be biomarkers showing physiological changes. The expression level of enzymes is closely related to some diseases, such as cancer and neurological diseases. Enzyme-based nanomaterials have unique advantages due to enzymes' nanoscale structure and biological catalysis functions. Biological endogenous enzymes can participate the bio-chemical reactions without external stimulation, and they have excellent biocompatibility, special recognition, and high biosafety. In addition, enzymatic reactions occur fast and are highly efficient in mild conditions. Various biological enzymes such as proteases, oxidoreductases, kinases, and others have been widely reported in the last years, which have exhibited great potential for the fabrication of functional enzyme-based nanomaterials [96].

Glucose oxidase (GOx), catalase (CAT), proteolytic enzymes, and other enzymes play an important role in cancer treatment. Enzyme-based nanotherapeutics overcome the shortcomings of general biological enzymes, such as low stability and short duration of action. For instance, Dang et al. synthesized a dual-nanozyme chemiluminescence (CL) system by combining natural GOx together with iron porphyrin MOF (FePorMOF) [97]. In the process, nanoscale GOx participated in the reduction reaction and the generation of H_2_O_2_, and then the generated H_2_O_2_ reacted on the surface of FePorMOF to generate CL. This in-situ generation of H_2_O_2_ via GOx significantly enhanced the luminescence time of the CL system (Fig. 8b).

In addition, enzyme immobilization is an important strategy to stabilize its conformation and catalytic activity. In a recent work, silica@CAT/ZIF-8 nanocomposites have been prepared successfully via biomimetic silicification using mesoporous silica to coat CAT-immobilized ZIF-8 [98]. Under the protection of the outer layer of mesoporous silica, CAT/ZIF-8 nanocrystals avoided the activity reduction or degradation due to changes in external conditions. After 10 cycles of use, a catalytic activity of 50% was still maintained, which greatly improved the service life of CAT enzyme.

In a similar study, Song et al*.* produced novel enzyme-based nanomaterials by encapsulating CAT inside tantalum oxide (TaOx) hollow nanoshells. They prepared TaOx@CAT nanohybrids, which were further functionalized with PEG to form functional TaOx@CAT-PEG nanomaterials with good biocompatibility [99]. It has reported that CAT decomposed intracellular H_2_O_2_ and produced O_2_; thereby, the created TaOx@CAT-PEG nanomaterials could overcome hypoxia-induced therapy resistance and improve the efficiency of the radiation therapy.

Finally, we present a table (Table 1) to summarize the material synthesis strategies and applications of BBNMs.

**Table 1:** Summary of synthesis and applications of biomimetic nanomaterials, especially in biomedicine

Biomolecule	Nanomaterials	Synthetic method	Application	Refs
DNA	DNA-Micellar nanoparticles	Molecule–molecule interactions	Treating eye infections	[54]
	Ca^2+^/(Alg/PEI/DNA) nanoparticles	Molecule–molecule interactions	Gene therapy for cancer	[57]
	DNA nanoparticles	Molecule–molecule/material recognition	Drug-resistance	[58]
	DNA nanoparticles	Molecule–molecule/material recognition	Tumor-targeted delivery	[59]
	DNA Nanofiber	Molecule-mediated nucleation and growth	–	[60]
	DNA Nanofiber	Molecule–molecule interactions	Traceable gene delivery	[61]
	DNA-Linked Nanoparticle Films	Molecule-mediated nucleation and growth	Traceable gene delivery	[62]
	Au NP-embedded SDNA thin films	Drop-casting method	UV photodetectors	[65]
	DNA-derived carbon dots	Hydrothermal synthesis	Electrochemical sensing	[67]
RNA	RNA triangular nano-scaffolds	Molecule–molecule interactions	Treating myocardial infarction	[71]
	RNA nanoparticles	Molecule–molecule/material recognition	–	[72]
Peptide	Exosome/PGN hydrogel	Molecule–molecule interactions	Treating myocardial infarction	[84]
	Nano micelles	Molecule–molecule interactions	Targeting drug delivery system for breast cancer treatment	[86]
Protein	CUR/PTX@RSF/zein nanospheres	Molecule-mediated nucleation and growth	Cancer treatment	[87]
	rGO/BSA-AuNC platform	Biomolecule-medicated oxidization/reduction process	Detection of activity and concentration of trypsin	[89]
PDA	PDA-PSB co-deposition surface coating	co-deposition	Anti-fouling coating of neural probes	[91]
	PDA-Pt nanocomposite	Biomolecule-medicated oxidization/reduction process	Catalysis	[95]
Enzyme	FePorMOF/Gox CL system	FeP/ C nanosheets	Chemiluminescence	[97]
	silica@CAT/ZIF-8 nanocomposites	–	Biocatalyst	[98]
	TaOx@CAT NPs	A one-pot method	Radiotherapy	[99]

## Biomolecule-mimetic nanomaterials for PTT and PDT applications

### BBNMs for PTT applications

As mentioned above, PTT is an emerging disease treatment method, in which a specific laser is used to illuminate the tumor site with a photothermal agent injected. This absorbs the light energy and converts it into released heat [100]. When the temperature reaches above 48 °C, cancer cells undergo necroptosis within minutes [101]. Compared with traditional cancer treatment methods, the effect of PTT can be modulated by adjusting the irradiation parameters of the laser. On the other hand, this method is considered non-invasive and is associated with quick recovery, significantly reducing the patient’s pain. [102].

PTT is a flexible treatment method that can eliminate a variety of tumor cells, and can also can be used as a supplement to surgery or chemotherapy, increasing cancer cure rates [103]. Although PTT has many advantages over traditional methods, it encounters many problems in clinical applications, in which many photothermal materials have disadvantages such as poor biocompatibility, long-term toxicity, and poor targeting [104]. In order to overcome these problems, the use of BBNMs as PTC agents has become a new prolific research direction.

### Peptide and protein nanomaterials for PTT

**Fig. 9:**
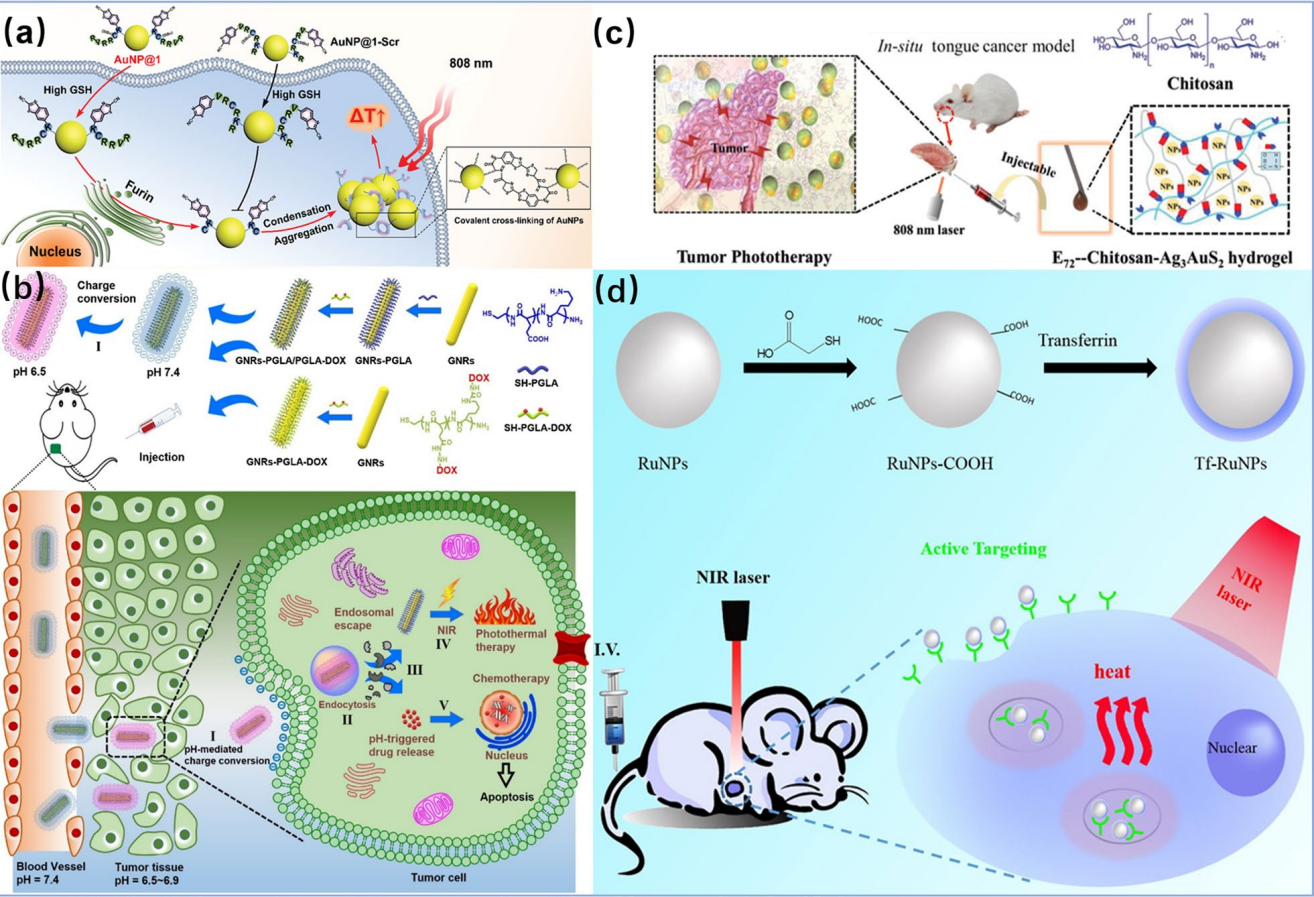
**a** Furin-directed aggregation of AuNPs in AuNP@1 cells for tumor PTT. Reprinted with permission from Ref. [106], Copyright 2020, Wiley–VCH. **b** preparation and progress of GNRs-PGLA/PGLA-DOX zwitterionic conjugates the process of chemo-photothermal therapy. Reprinted with permission from Ref. [107], Copyright 2021, Elsevier. **C** the process of E72-chitosan-Ag_3_AuS_2_ treatment of tongue tumor in situ. Reprinted with permission from Ref. [109], Copyright 2021, Wiley–VCH. **d** the preparation of Tf-modified RuNPs and the process of PTT. Reprinted with permission from Ref. [111], Copyright 2018, Elsevier

Peptide-mimetic nanomaterials are widely used in biomedicine due to their structural and functional diversity, tunable behavior, high biocompatibility, and good biodegradability [105]. Recently, Chen et al*.* designed an AuNP-responsive platform (AuNP@1) modified with a specific peptide sequence, which can induce the aggregation of AuNPs by furin in cancer cells [106]. Furin is present in a variety of malignant tumor cells. According to the property of furin to limit the cleavage of Arg-X-Lys/Arg-Arg peptides, a 2-cyanobenzothiazole (CBT) motif was used to link the peptide-modified AuNPs. When AuNP@1 entered into the cancer cells, the CBT-Cys condensation reaction occurred after the reduction of intracellular reduced glutathione (GSH) and the cleavage of the polypeptide by furin, causing the aggregation of AuNPs in the cancer cells (Fig. 9a). To prove the effect of peptide-modified AuNPs, an AuNP@1-Scr without AuNP aggregation platform was designed as a control experiment. The survival rate of cancer cell with 400 µg mL ^−1^AuNP@1 was 25%, which was 45% lower than that of the AuNP@1-Scr group at the same concentration. In vivo, the local tissue temperature in the AuNP@1 group was up to 44.9 °C, which was 10 °C higher than that of the AuNP@1-Scr group. It is clear that the cancer cells treated by AuNP@1 + laser irradiation mostly died. After 18 days of light treatment, the tumor growth of the AuNP@1 group was significantly inhibited compared with the control group, and the body weight of the mice did not fluctuate significantly during the treatment period. This study proves that the peptide-modified AuNP@1 therapeutic platform has high performance and excellent biocompatibility.

In another work, Huang et al. used a pH-triggered charge-switching amphiphilic polypeptide thiol-terminated poly(l-Glu-*co*-Lys acid) (SH-PGLA) to form a conjugate with GNRs and linked DOX to the polypeptide through a hydrazone bond to construct a GNRs-PGLA/PGLA-DOX zwitterionic GNRs conjugate. Under physiological pH conditions, the surface of the amphiphilic polypeptide maintains a negative charge, which prevents binding to serum proteins and restriction endonuclease (REC) and prolong its half-life. The weakly acidic (pH = 6.5–6.9) surface of the amphiphilic polypeptide around the cancer cells is converted into a positive charge, thus promoting the uptake of the zwitterionic GNR conjugates by the cancer cells. Since the pH of intermediate and advanced cancer cells is below 5.0, the hydrazone bonds on the GNR conjugates are broken under acid conditions to release DOX. This achieves the purpose of a combined chemo-PTT (Fig. 9b). Compared with single chemical therapy or single PTT, the inhibition efficiency of cancer cells was increased by 20%, and the peptide as a carrier improves the biocompatibility of the photothermal agents [107]. Nanomaterials constructed with peptides significantly improve the stability, targeting, and biocompatibility of photothermal agents.

Proteins have excellent biocompatibility and biosafety, are ideal biomolecules for the formation of biomimetic nanomaterials. They can be combined with many inorganic materials, exhibit different physicochemical properties and have wide applications in the biomedical field [108]. For instance, Su et al. prepared an injectable hybrid protein hydrogel (E72-Chitosan-Ag_3_AuS_2_NP) by genetically engineering anionic protein, chitosan, and Ag_3_AuS_2_NPs together [109]. The complexation of Ag_3_AuS_2_NPs with proteins in the hydrogel can significantly reduce its cytotoxicity and improve its biocompatibility and stability (Fig. 9C). In the experiment, it can be clearly observed that the tumor mass of affected mice treated with the protein hybrid hydrogel disappeared, and their tongue function, previously impaired by the tumor, recovered.

Wang and co-workers used BSA as a chelating agent to synthesize BSA-Ag_2_SNPs at mild temperature [110]. The combination of BSA molecules and Ag_2_S NPs formed biomimetic nanohybrids with good water solubility and high stability. No significant aggregation occurred after one month of incubation in PBS buffer solution. In the in vitro experiments, the survival rate of HeLa cells irradiated with 808 nm laser for 5 min was less than 25%. The tumor volume of mice in the experimental group gradually decreased within 5 days of PTT, and eventually eliminated. It was proved that BSA-Ag_2_S NPs have a good PTT efficacy. In another case, Zhao et al*.* used transferrin (Tf) to modify ruthenium (Ru) NPs to improve the biocompatibility and cellular uptake of Ru NPs by cells [111]. There is a large number of transferrin receptors (TfR) on the surface of cancer cells. Studies have shown that Tf/TfR-recognition-mediated endocytosis could effectively promote the aggregation of NPs in tumor cells, which greatly improved the selectivity of NPs to tumor cells (Fig. 9d). ICP-MS was used to detect the uptake of both Tf-modified and unmodified Ru NPs by cancer cell A549, and it was found that the uptake rate of Tf-RuNPs was three times higher than that of RuNPs. These results demonstrate that the modification of Tf greatly enhances the uptake of Ru NPs by cancer cells. Compared to the same concentration of AuNPs in human embryonic kidney, MTT measurements in 293 cells measured showed that the cell survival rate under the condition of 200 μgmL^−1^ Tf-RuNPs was 82.2%, compared with 38.7% under the same concentration of GNRs. In the in vitro PTT experiment, the cell survival rate was only 18.3% after the irradiation of the mixed Tf-RuNPs and cancer cells A549 with 808 nm laser for 1 min, and the cell survival rate was decreased to 3.1% quickly after 2 min. The modification of photothermal agents with protein biomolecules can therefore effectively improve their stability and biocompatibility, and play an important role in realizing PTT with low side effects.

#### PDA nanomaterials for PTT

**Fig. 10:**
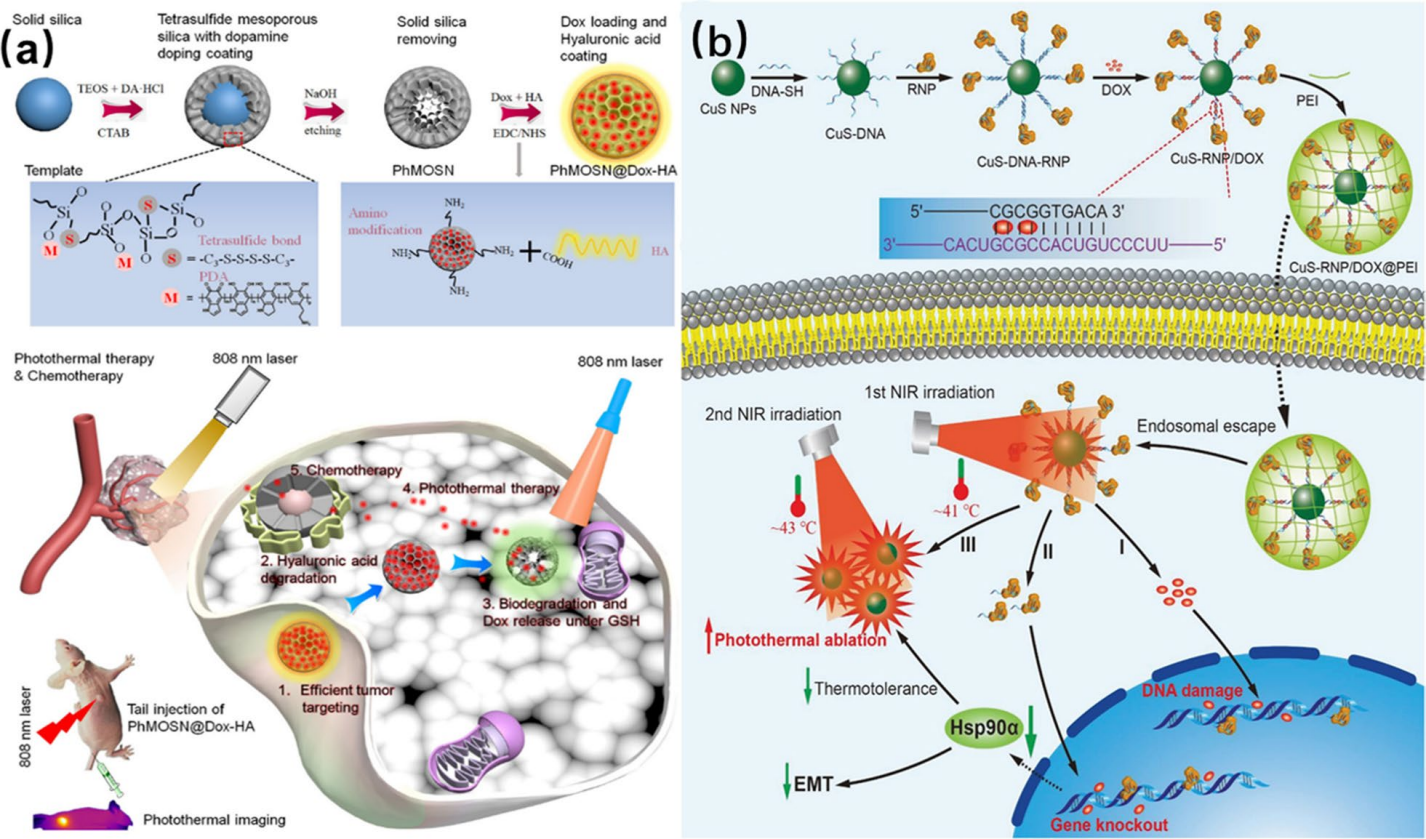
**a** Tumor synergistic chemo-photothermal therapy process of PhMOSN@DOX-HA. Reprinted with permission from Ref. [114], Copyright 202, Elsevier. **b** Preparation of CuS-RNP/DOX@PEI nanotherapeutic platform and photothermal-chemical synergistic treatment process. Reprinted with permission from Ref. [119], Copyright 2021, Wiley–VCH

The function of PDA-based nanomaterials is controlled by its concentration, size and the solution pH in which they are dispersed [112, 113]. For instance, Hu et al*.* used a facile method to prepare an organosilicon PDA framework bonded to GSH-sensitive hollow tetrasulfide and co-doped with mesoporous silica nanospheres (PhMOSN) [114]. DOX were loaded in pores inside the framework and the whole particles were coated with hyaluronic acid (HA). Finally, a targeted breast cancer therapeutic platform (PhMOSN@DOX-HA) was formed (Fig. 10a). The biocompatibility, biodegradability, and PTT of the platform were evaluated. After incubation of 300 μg/mL PhMOSN@DOX-HA with common breast cells for 24 h, 90% of the cells survived. This demonstrated that the PhMOSN@DOX-HA-based breast cancer therapeutic platform exhibited good biocompatibility. In addition, PhMOSN was substantially degraded within two hours at 10 mM GSH concentration, proving that the platform had good biodegradability. After irradiating PhMOSN with 808 nm laser for 5 min, the photothermal temperature increased to 55 °C and its PTC efficiency was calculated to be as high as 54.08%.

In another case, Wang et al*.* coated molybdenum selenide (MoSe_2_) nanosheets with PDA, and then the PDA layer was loaded with the anticancer drug DOX to form a MoSe_2_@PDA-DOX capacitive nanotherapeutic platform with high biocompatibility [115]. The HeLa cells viability was decreased to 17% after treatment with MoSe_2_@PDA-DOX and laser-irradiation. Also using PDA as a coating and photothermal conversion material, Zhang et al*.* used self-assembly to encapsulate DOX in nano-mesoporous ZIF-90 to obtain ZIF-90@DOX. Then they used a hard-templating method to encapsulate ZIF-90@DOX[116]. PDA@DOX NPs for breast cancer were prepared by coating DOX with PDA. The DOX loading of the nano-therapeutic drug reached 53.16%. When the temperature of the nanoparticles increased under laser irradiation, the DOX was released. Therefore, the good laser sensitivity of PDA@DOX NPs can greatly improve the effect of drugs and reduce the damage to normal cells during chemotherapy. In subsequent in vivo experiments, mice treated with PDA@DOX NPs maintained a stable body weight compared to mice treated with DOX alone, and tumor cells almost disappeared after 16 days. The experiments strongly demonstrate the superiority of PDA@DOX NPs for tumor therapy.

#### DNA and RNA nanomaterials for PTT

Gene therapy is an important technique for cancer treatment, which uses DNA and RNA to alter the expression of deregulated genes to achieve the purpose of treatment. Gene therapy and PTT can be combined to treat specific cancers with good therapeutic effects [117, 118]. For example, Chen et al*.* established a CuS-RNP/DOX@PEI nanotherapeutic platform that can release Cas9 RNP composed of clustered regularly interspaced short palindromic repeat (CRISPR)-CRISPR associated 9 (Cas9) nuclease and single guide RNA (sgRNA) by PTT, and simultaneously release DOX embedded in DNA to achieve a synergistic-release effect of chemotherapy [119]. As shown in Fig. 10b, the specifically designed DNA (DNA-SH) fragment is modified by thiol to bind to CuS NPs, and then the Cas9 RNP was connected to DNA by the principle of base complementarity to form a complementary double strand (DNA-SH/sgRNA). DOX was inserted into DNA-SH/sgRNA and finally coated with endosome-disrupting polyethyleneimine (PEI) to facilitate cellular internalization. When the platform enters the cell interior, PEI ruptures to release CuS-RNP/DOX, and under the induction of NIR laser, CuS undergoes PTC, the temperature raises to about 41 °C. In turn, this induces the breakage of complementary double-strand (DNA-SH/sgRNA) to release Cas9 RNP with DOX. On the one hand, Cas9 RNP inhibits the expression of Hsp90 protein in cancer cells to reduce the heat tolerance and metastatic ability of cancer cells to achieve the purpose of gene therapy. Meanwhile, the release of DOX inside the cells achieved the purpose of chemotherapy. Then, the PTC ability of CuS was fully exerted by a second laser irradiation, and the cancer cells were ablated under milder temperature conditions (43 °C). In the in vivo experiments, the tumors of the mice treated with CuS-RNP/DOX@PEI were almost completely eliminated.

DNA molecules can be highly cross-linked by self-assembly or chemical cross-linking to form hydrogels with excellent physical and chemical properties [120]. Liu used DNA electrostatic complexation to upconvert lanthanide-Au hybrid nanoparticles (UCNP-Au NPs) to prepare novel injectable DNA-inorganic hybrid hydrogel with PTC properties [121]. Compared with general inorganic hybrid nanomaterials, DNA-UCNP-Au exhibited higher biocompatibility and PTC efficiency. It could be effective at the tumor site through local precision treatment, and had no toxic side effects on normal tissue cells. Under 1 W·cm^−2^ 808 nm laser irradiation towards 0.2 w% UCNP-Au NPs, the temperature was increased to as much as 87 °C within 3 min, and the PTC efficiency reached 42.7%, which was 10% higher than that of UCNP-AuNPs. More than 70% of cancer cells were killed after irradiating the DNA-UCNP-Au hydrogels for three minutes with infrared laser. In the mouse experiments, the upconverted emission signal confirmed that UCNP-Au was decomposed within a few hours, while the DNA-UCNP-Au hydrogel remained intact for 24 h. After 21 days of treatment, the mouse cancer cells treated with DNA-UCNP-Au hydrogel were basically eliminated and no toxic side effects were produced on the normal tissue cells. The above results prove that DNA-biomimetic hydrogels have excellent PTC ability and biological stability in organisms, and can achieve efficient removal of cancer cells through local drug injection.

#### Enzyme nanomaterials for PTT

Compared with natural enzymes, enzyme-mimetic nanomaterials have higher biostability and biocompatibility, and lower cost [122]. Specific enzyme nanomaterials also have certain targeting properties and have excellent effects in the treatment of certain cancers [123].

**Fig. 11:**
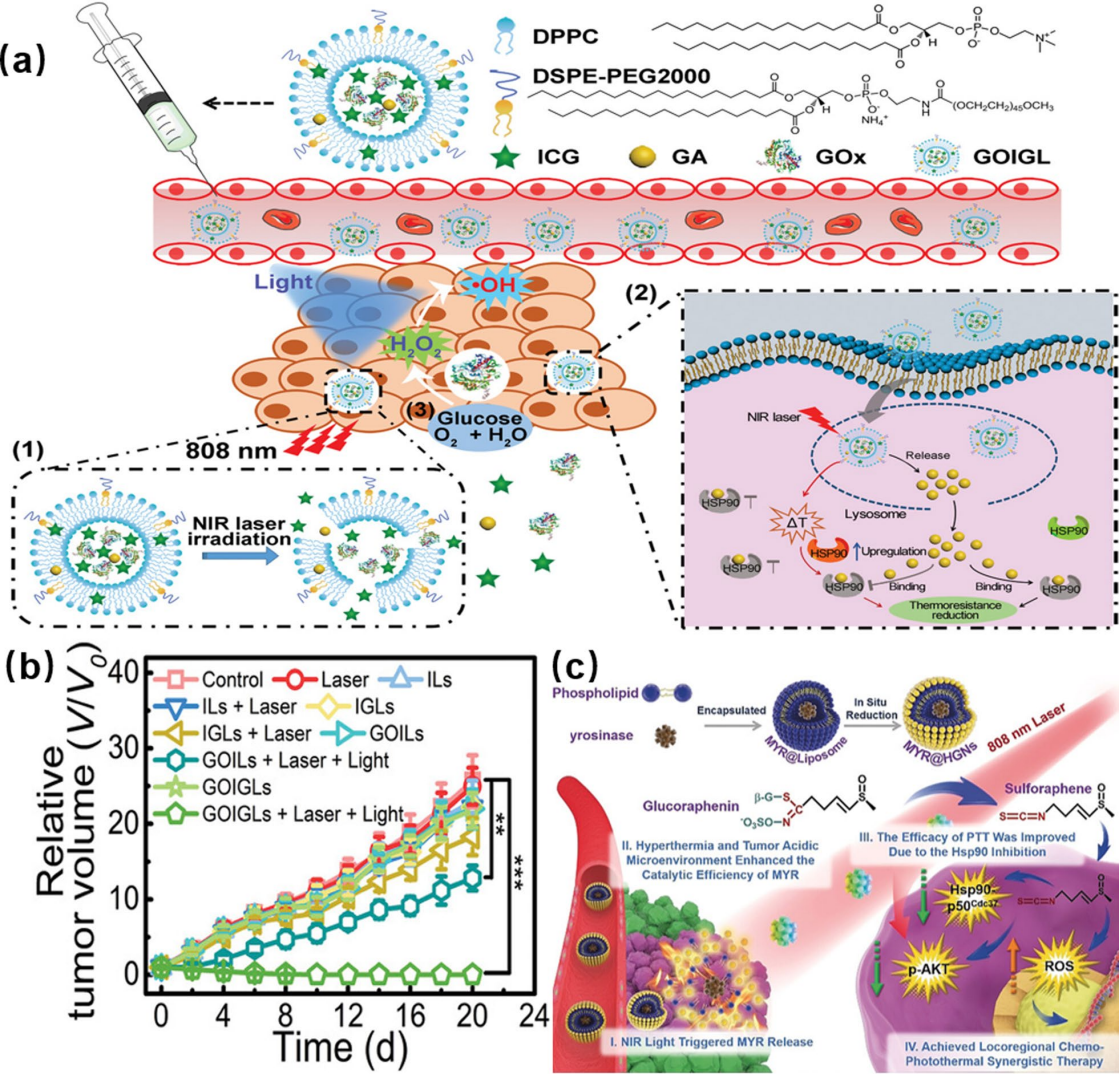
**a** The preparation of GOIGL and the process of synergistic starvation/photothermal therapy. **b** time and relative tumor volume curves of tumor-bearing mice treated with different conditions. Reprinted with permission from Ref. [124], Copyright 2016, Wiley–VCH. **c** preparation and chemistry of MYR@HGN for PTT. Reprinted with permission from Ref. [127], Copyright 2019, Wiley–VCH

Gao et al. used 1,2-dipalmitoyl-sn-glycero-3-phosphocholine (DPPC) and 1,2-distearoyl-sn-glycero-3-phosphoethanolamine-N-[methoxy(polyethylene glycol)-2000] (ammonium salt) (DSPE-PEG2000) to coat GOx, indocyanine green (ICG), and gambogic acid (GA) to prepare thermosensitive GOx/ICG/GA liposomes (GOIGLs) [124]. Laser irradiation triggers the photothermal conversion effect of indocyanine green (ICG), which increases the temperature. Thermosensitive GOx/ICG/GA liposomes (GOIGLs) release GOx, ICG, and GA. The released GOx can effectively catalyze the oxidation of glucose in cancer cells and generate H_2_O_2_, thereby limiting the supply of ATP and inhibiting the expression of heat shock proteins (HSPs) to reduce the heat resistance of cells. The oxidative H_2_O_2_ can be converted into hydroxyl radicals (·OH) under light exposure to cause oxidative damage to cancer cells. In addition, GA can bind to the heat shock protein HSP90 and inhibit its activity, further reducing the heat resistance of cancer cells, and achieving photothermal therapy at mild temperatures (Fig. 11a). In 10 groups of mice experiments, the ablation effect of cancer cells in the “GOIGLs + Laser + Light” group was significant (Fig. 11b). Compared with PBS-treated mice, the maximum temperature in the tumor area did not exceed 43 °C after GIOGL suspension (1 mg/kg ICG). After the treatment, the body weight changes of the mice were compared and the main organs (heart, liver, spleen, lung and kidney) of the mice were dissected and compared with the mice in the PBS group. No obvious organ damage was found, indicating that GOIGLs have good biocompatibility and neglectable toxicity. In another work, Liu et al. used GOx to enhance cytochemical kinetics and PTT performance [125]. Fe_3_O_4_ NPs were firstly modified with polyethylene imine (PEI) and the, reacted with multi-walled carbon nanotubes (MWNT) and PEG to form PEG-MWNT-Fe_3_O_4_ (PMF). The PMF-GOx (PMFG) composite material was prepared by combining GOx with PMF via amide bond formation. In the process of treatment, GOx in PMFG converted glucose into H_2_O_2_ and provided reactants for the subsequent Fenton reaction of Fe_3_O_4_ to produce a large amount of •OH. In addition, CNTs generated heat rise under the irradiation of a near-infrared laser. The irradiated composite material produced thermal ablation of tumor cells and enhanced the Fenton reaction. The synergistic effects of both components not only increased the yield of •OH, but also improved the therapeutic effect on tumors.

Most recent studies focus on using nanoparticles for catalyzing chemical reactions and for the thermal ablation of cancer cells. The use of nanoparticles to immobilize enzymes at first to control their catalytic activity and then increase the heat to produce its thermal effect is still under development.

Xu et al*.* also used GOx to modify N-doped carbon (NC) NPs to obtain multifunctional NC@GOx NPs [126]. The biomimetic NC@GOx NPs exhibited synergistic effects of GOx-dominated starvation, chemotherapy, and carbon NP-dominated PTT. Cheng et al. used a lipid membrane hydration method to encapsulate myrosinase (MYR), an exogenous enzyme, in the water cavity of liposomes, which was then coated with GNR to obtain a MYR@HGNs therapeutic platform [127]. The MYR release was activated using NIR laser (808 nm) after MYR@HGNs entered cancer cells. The released MYR can convert glucosamine (GRE) to toxic sulforaphane (SFE, an isothiocyanate) within cancer cells. The obtained results indicated that SFE can induce apoptosis of cancer cells by increasing the level of reactive oxygen species (ROS) on the one hand. On the other hand, it can inhibit the function of Hsp90 and improve the therapeutic effect of PTT (Fig. 11c). In the animal experiment, it was found clearly that the MYR@HGNs + GRE + NI group-treated mice made the temperature to increase to 56.3 °C within 5 min, and the tumor cells were completely eliminated after 16 days of treatment.

### BBNMs for PDT applications

PDT is a new type of disease treatment method with great development prospects. As a non-invasive method, it has a good therapeutic effect in tumor treatment [128]. In the process of PDT, ROS are generated under specific light conditions based on non-toxic photosensitizers to destroy cellular structures such as target proteins and nucleic acids, and further induce apoptosis. Although PDT has many advantages, there are still some shortcomings that hinder its development. For example, traditional photosensitizers have poor water solubility, high cytotoxicity, poor targeting, and low biocompatibility, and may induce cellular hypoxia during treatment, which greatly reduces the therapeutic effect [129]. With the development of biotechnology, biomolecules were used to augment and construct photosensitizers to compensate for the shortcomings of PDT treatment, which is one of the most important directions to further improve the therapeutic effect of PDT.

#### Peptide and protein nanomaterials for PDT

Using the self-assembly and target recognition functions of peptides to design PDT nanomedicines is an effective way to improve the effect of PDT [130]. Wu et al*.* designed a drug delivery nano-platform (cRGD@TAT-DINPs) based on TAT and cRGD peptides, which realized the co-delivery of DOX and indocyanine green (ICG) [131]. Under the triggering of NIR light, they achieved the purpose of synergistic chemo/photothermal/photodynamic therapy. Their results indicate that the addition of functional peptides can significantly improve the therapeutic effect of nano-drugs, and the bioavailability and targeting of drugs have a huge impact on their therapeutic effects. In another work, Yan et al. used a cancer cell-targeting CD133 peptide (LQNAPRS) coupled with pyropheophorbide-a (Pyro) to prepare a PDT photosensitizer with cancer-cell-targeting properties [132]. HT29 cells with high expression of CD133 can be efficiently targeted within 1 h at a final concentration of 1 μM at 4 °C. In the mouse experiment, 2.25 mg kg^−1^ of CD133-Pyro was injected into mice, and the fluorescence signal of D133-Pyro was mainly concentrated in the tumor area within 9–24 h of injection. The obtained results fully proved that the PDT with CD133-Pyro as a photosensitizer had an obvious antitumor effect on colorectal cancer (CRC) and cancer stem cells (CSC)-derived tumors. The combination of polypeptide molecules and photosensitizers not only gave them a high degree of targeting, but also promoted a synergistic therapeutic effect.

**Fig. 12:**
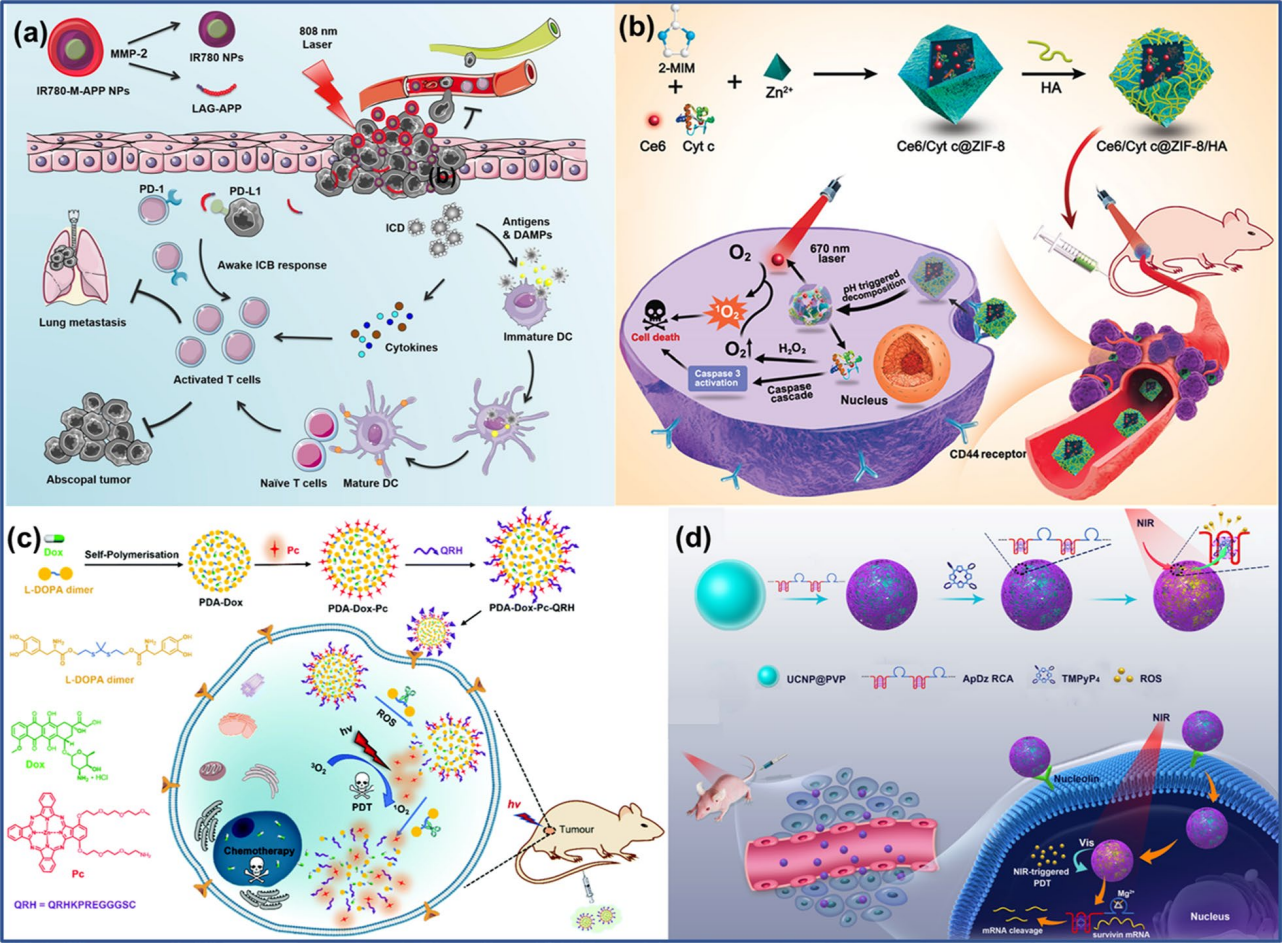
**a** Photodynamic immunotherapy action process of IR780-M-APP NPs Reprinted with permission from Ref. [134], Copyright 2021, Elsevier. **b** Synthesis of Ce6Cyt c@ZIF-8HA NPs and their targeted photodynamic and protein synergistic therapy process. Reprinted with permission from Ref. [135], Copyright 2020, American Chemical Society. **c** PDA-Dox- Pc-QRH preparation and photodynamic and chemical synergistic treatment process. Reprinted with permission from Ref. [138], Copyright 2021, Royal Society of Chemistry. **d** Synthesis and DNA/polymer hybrid materials for cancer PDT. Reprinted with permission from Ref. [137], Copyright 2021, Wiley–VCH

Clinical experiments have shown that immune checkpoint antibodies have a significant effect on enhancing anti-tumor immunity [133]. For instance, Wang et al*.* developed a programmed death-ligand 1 (PD-L1) peptide (APP, NYSKPTDRQYHF-NH_2_) to replace the PD-L1 antibody, and constructed a multifunctional photodynamic immunotherapy nano-system by combining APP together with the photosensitizer IR780 [134]. IR780-M-APP NPs are highly targeted, and the tumor fluorescence intensity of IR780-M-APP NPs in mice is much stronger than that of free IR780 at the same time. On the other hand, IR780 generated by IR780-M-APP NP is degraded after internalization by tumor cells, and generates ROS to induce apoptosis under specific laser irradiation (Fig. 12a). In subsequent in vivo experiments in mice, the IR780-M-APP NPs-treated group showed a higher inhibitory effect than the control group, and the synergistic therapeutic effect of photodynamic and immunotherapy was obvious. The multifunctional nanodrug design strategy in this work provides a solution for synergistic cancer therapy using peptide molecules.

Ding et al. reported a co-delivery nanoplatform for cancer cells targeting photosensitizers and therapeutic proteins based on MOF [135]. As shown in Fig. 12b, chlorin e6 (Ce6) and cytochrome C (Cyt c) are encapsulated on ZIF-8 by a mild one-pot biomimetic mineralization method. The formed NPs are then covered with the cell-targeting ligand hyaluronic acid (HA) to prepare a tumor-targeting nanoplatform (Ce6/Cyt c@ZIF-8/HA). After Ce6/Cyt c@ZIF-8/HA is absorbed by cancer cells through the targeting effect of HA, Ce6 and Cyt c are released under the action of acidic environment and cell lysosomes. Under laser irradiation, Ce6 can produce cytotoxic ^1^O_2_ to kill tumor cells. Meanwhile, Cyt c can induce programmed cell death, and under hypoxic conditions, Cyt c can maintain the catalase-like function to decompose H_2_O_2_ to generate O_2_, and relieve hypoxia during the PDT process. Both in vivo and in vitro treatment experiments demonstrated that Ce6 and Cyt c have obvious synergistic therapeutic effects, and have excellent biocompatibility and targeting.

In another work, Chen et al*.* combined human serum albumin (HSA) with hemoglobin (Hb) via intermolecular disulfide bonds to form biomimetic hybrid protein oxygen nanocarriers, which were further used to load chlorine e6 (Ce6) to form a PDT nanomaterial (C@HPOC) [136]. The method of hybridization with HSA makes up for the shortcomings of short circulation time and poor stability of Hb, improves its biocompatibility, and exerts its ability to run oxygen with targeting. The experiments demonstrated that the oxygen supply of Hb greatly enhanced the generation of ROS, and the viability of tumor cells treated with C@HPOC + laser dropped to 11%, which was 7 times lower than that of cells treated with Ce6 + laser and C@HSA + laser.

Li et al*.* used BSA to encapsulate Pt NPs to form Pt NCs, which were then modified with folic acid (FA)and combined with methylene blue(MB)-loaded mesoporous silica nanospheres (MSNS) as a biocoating to prepare the PDT nanopolymers (FA/PtBSA@MB-MSNS) with high targeting and continuous oxygen supply abilities [137]. The as-prepared biomimetic nanomaterials have the following characteristics: (i) they only release ROS in cancer cells, which is highly targeted; (ii) the addition of BSA protects the catalytic activity of Pt nanoclusters in complex cellular environments; (iii) BSA as a coating for nanomaterials ensures its good biocompatibility and reduces the toxic side effects to normal cells. In the experimental group of tumor mice with the created nanomaterials, Pt catalyzed the conversion of intracellular H_2_O_2_ to O_2_, which improved the effect of tumor local hypoxia on PDT and greatly improved the therapeutic effect.

#### PDA nanomaterials for PDT

PDA can also be used as a nano-coating to modify various materials for PDT applications. Dai et al. designed highly sensitive ROS-responsive PDA nanocarriers that can react with trace amounts of ROS in tumor cells to release chemotherapeutic drugs and photosensitizers [138]. Meanwhile, ROS generated by photosensitizers can further promote the release of therapeutic drugs and photosensitizers from PDA NPs, and also promote the degradation of PDA NPs. As shown in Fig. 12c, DOX-encapsulated PDA NPs were used to link a di-α-substituted Zn^2+^ phthalocyanine (Pc) based photosensitizer molecule and a QRHKPRE peptide sequence that can target the epidermal growth factor receptor (EGFR) overexpressed in cancer cells, and finally multifunctional PDA-DOX-Pc-QRH NPs were obtained. After selective uptake of PDA-DOX-Pc-QRH NPs by EGFR-positive cancer cells, thioketal bond cleavage under the action of ROS in the tumor microenvironment released DOX and Pc molecules. Pc molecules were subsequently activated under specific laser irradiation to generate ROS, which acted as PDT and further promoted the cleavage of PDA-Dox-Pc-QRH NPs. Meanwhile, DOX molecules were released inside cancer cells for targeted chemotherapy. PDA-Dox-Pc-QRH NPs showed excellent antitumor effect through synergistical PDT and chemotherapy in this study.

In another work, Wang et al. used MnO_2_ modified with an RGD-functional polymer to coat hollow PDA NPs, and further used polyethylene glycol (PEG) to load the photosensitizer Ce6 and the chemotherapeutic drug DOX to obtain a PDA@MnO_2_@Ce6/DOX@PEG-RGD multifunctional therapeutic nanoplatform [139]. This therapeutic nanoplatform can reach the tumor cell site through the targeted recognition function of RDG, and PDA decomposes and releases Ce6 and DOX in the slightly acidic environment of the tumor for combined photodynamic and chemical therapy. MnO_2_, released at the same time, can convert the endogenous H_2_O_2_ of tumor cells into O_2_ to overcome the hypoxia of PDT treatment and greatly improve the therapeutic effect of PDT.

#### DNA and RNA nanomaterials for PDT

DNA is a flexible and programmable nanomaterial [140]. A multifunctional up-conversion nanoplatform (UCNP-ApDz-TMPyP_4_) was designed for combining PDT with gene therapy, whereby an aptamer-DNAzyme (ApDz) was adsorbed via electrostatic interactions [141]. As shown in Fig. 12d, the aptamer (AS1411) can load the photosensitizer 5,10,15,20-tetrakis(1-methylpyridinium-4-yl) porphyrin (TMPyP_4_), and meanwhile it can recognize nucleolin expressed intracellularly in cancer cells. The encoded ssDNA can effectively reduce the surviving gene expression, which can improve the therapeutic effect of PDT. The growth of tumors treated with UCNP-ApDz-TMPyP_4_ was significantly inhibited. Tumor cells were stained with hematoxylin and eosin (H&E) and terminal deoxynucleotidyl transferase-mediated UTP end labeling (TUNEL) revealed that UCNP-ApDz-TMPyP_4_ resulted in increased apoptosis and obvious pyknosis, nuclear rupture, and cell necrosis.

Pan et al*.* constructed a DNA nanosponge by encoding specific sequences and integrating multiple functions, which could load photosensitizers and target tumor cells for efficient PDT therapy [142]. In this work, a sgc8c aptamer that can target cancer cells overexpressing protein tyrosine kinase 7 (PTK-7) was designed as a G-quadruplex that can intercalate the porphyrin photosensitizer TMPyP4, and can enhance PDT sensitivity. The HIF-1α antisense DNA complementary sequence and the incorporation of catalase during the reaction were assembled by RCA to obtain a multifunctional DNA nanosponge with good biocompatibility. The antitumor efficacy and biocompatibility of DNA assemblies were evaluated using HeLa tumor-bearing mice. The distribution of Cy5-labeled DNA assemblies was observed. After six hours of injection, obvious fluorescent signals were found in the tumor area. After laser irradiation treatment, it was found that the tumor growth was significantly inhibited compared with the control group. Significant decreases in the expression of HIF-1α and VEGF were found in the DNA assembly group in tumor sections from mice, suggesting that the DNA assemblies could be effective against hypoxia-related photodynamic resistance during PDT in vivo. Finally, the blood biochemical information and tissue sections of the mice after 14 days of treatment showed no obvious abnormality and tissue damage, indicating that the DNA assembly had good biocompatibility.

RNA interference (RNAi) is a gene therapy method that has been widely studied, mainly through the addition of exogenous RNA to achieve targeted gene silencing[143]. Zhang et al. designed a compound composed of PEGylated-branched polyethylenimine (PEI), 3,3′-(propane-2,2-diylbis(sulfanediyl)) dipropanoic acid (ROS-cleavable linker), photosensitizer Ce6 and targeted the tumor cancer Cell proliferation gene RRM2/siRNA complex (PPTC/siRNA). Under the irradiation of near-infrared light, the PPTC/siRNA complex generates ROS, which leads to the degradation of the ROS cleavage linker and the release of siRNA. On the one hand, the released siRNA enters tumor cells to target and silence RRM2, reducing the proliferation of tumor cells; on the other hand, the ROS triggered by NIR causes tumor cell apoptosis. The combined treatment of PDT and RNAi greatly improved the anti-tumor efficiency, which provides a direction for novel cancer treatment strategies[144].

#### Enzyme nanomaterials for PDT

**Fig. 13:**
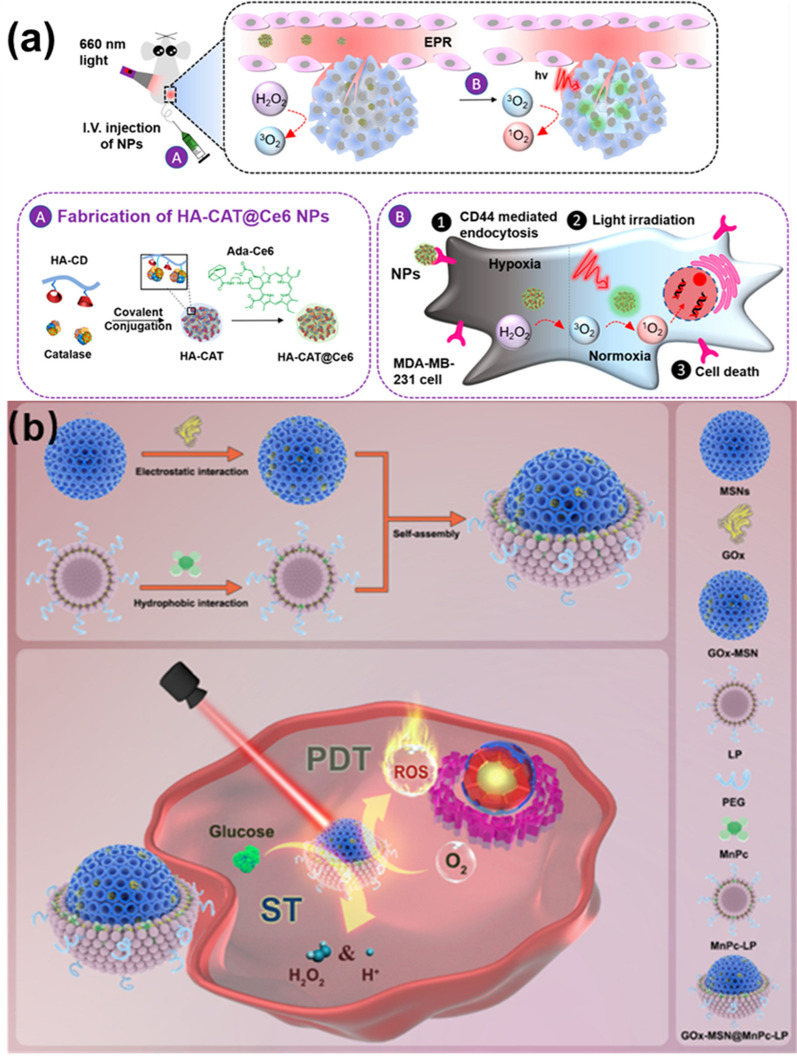
**a** Oxygen-enhanced photodynamic therapy process of HA-CAT@aCe6 NPs. Reprinted with permission from Ref. [146], Copyright 2019, American Chemical Society. **b** design strategy of GOx-MSN@MnPc-LP nanoreactor and photodynamic starvation synergistic therapy process in tumor cells. Reprinted with permission from Ref. [147], Copyright 2019, American Chemical Society

Various enzymatic biomimetic nanomaterials have been used in PDT to enhance the photo-PDT effect, such as catalase (CAT) and GOx, which can synergize PDT by modulating the tumor oxygen content and depleting intratumor glucose [145]. Soo et al. designed a nanotherapeutic system (HA-CAT@aCe6) consisting of a CD44-targeted HA, CAT, and adamantane-modified Chlorin e6 (aCe6) photosensitizer [146], as shown in Fig. 13a. HA can target the overexpressed CD44 receptor in tumor cells, increase the uptake of HA-CAT@aCe6 by tumor cells, and then under specific laser irradiation, aCe6 can generate ^1^O_2_ as a photosensitizer to destroy tumor cells. Meanwhile, CAT can catalyze endogenous H_2_O_2_ to generate H_2_O and O_2_ to overcome tumor cell hypoxia during PDT treatment and improve its therapeutic effect. In the experiment, the distribution of HA-CAT@aCe6 in mice 24 h after injection was observed, and it was found that the brightest fluorescence signal of HA-CAT@aCe6 appeared in the tumor site, whereas the fluorescence signal displayed in other major parts such as the liver was weak, indicating that HA-CAT@aCe6 showed highly targeting ability. In the in vivo PDT experiment, it was found that the tumor growth of the mice treated with HA-CAT@aCe6 was significantly inhibited, and the body weight of the mice did not decrease significantly during the whole experiment.

The main functions of enzymatic biomolecules in PDT applications are, on one hand, the use of the catalytic activity of enzymes to catalyze the production of O_2_, to enhance the effect of photodynamic therapy, and on the other hand, the applications mainly focus on decomposing glucose in cancer cells and producing toxic substances to destroy them. In a typical work, Zhu et al. loaded hydrophilic GOx into the pores of mesoporous silica nanoparticles (MSN) to form GOx-MSN, and meanwhile loaded hydrophobic manganese phthalocyanine (MnPc) onto liposomes to form MnPc-LP. The combination between Gox-MSN and MnPc promoted the formation of the GOx-MSN@MnPc-LP nanoreactor [147], as shown in Fig. 13b. After tumor cells internalize the nanoreactor, MnPc can generate ROS in under laser irradiation to destroy them, and GOx can continuously consume glucose inside the cells and generate toxic H_2_O_2,_ achieving high synergistic efficiency of starvation therapy and PDT. In the mouse experiment, after two weeks of treatment, it could be clearly seen that the tumors of mice treated with the biomimetic nanoreactors and laser irradiation completely disappeared after four days, indicating that the created GOx-MSN@MnPc-LP can elicit excellent therapeutic effect with low systemic toxicity and biocompatibility.

In a similar study, Fan et al. designed a targeted nanogel for self-oxygenated PDT of tumors [148]. Porphyrin and cancer cells targeted RGD peptide were aggregated as monomers into the nanogel and GOx and CAT were added to the gel for the formation of functional enzymatic nanomaterials. In this case, the addition of RGD peptide into the formed nanogel improved the targeting of materials to tumor cells. In addition, GOx promoted the biological catalysis of nanogel to convert glucose to glucose acid and H_2_O_2_, creating the hunger treatment effect. Meanwhile, CAT could decompose endogenous H_2_O_2_ and the GOx-induced H_2_O_2_ into H_2_O and O_2_. Under the treatment of PDT, the produced O_2_ could be transformed into ^1^O_2_, which can further improve the therapy efficiency of PDT. This strategy of enzyme-mediated combined starvation therapy and PDT provides a new idea on the development of nanomedicine for tumor therapy.

Here we provide a table (Table 2) to summarize the BBNMs for both PTT and PDT applications introduced and discussed above.

**Table 2:** Summary of biomolecule-mimetic nanomaterials for PTT and PDT applications

Biomolecule	Nanomaterials	method	Advantage	Type of cancer cell	
Peptide	AuNPs	PTT	Enhances the PTT efficiency of gold nanoparticles for furin-related cancer	MDA-MB-468 cell	[106]
	pH-sensitively zwitterionic polypeptide conjugated GNRs	PTT	Excellent biocompatibility against normal and cell pH-sensitively released the drugs under tumoral acid condition	HeLa cells	[107]
	cRGD@TAT-DINPs	CT/PTT/PDT	Excellent biological stability, combining multiple treatment methods	MDA-MB-231 and A549 cell	[131]
	CD133-Pyro	PDT	More effective targeted and intraocyric accumulation effects	Colorectal cancer cells	[132]
	IR780-M-APP NPs	PDT/immunotherapy	Targeted tumors and eradicated the metastatic and invasive tumors effectively	B16F10 cell	[134]
Protein	E72-Chitosan-Ag3AuS2 hybrid hydrogel	PTT	High biocompatibility and photothermal conversion efficiency	Tongue cancer cell	[109]
	BSA-Ag_2_S NPs	PTT	Biocompatibility	HeLa cells	[110]
	Tf-RuNPs	PTT	Biocompatibility and stability	HEK-293 cell	[111]
	Ce6/Cyt c @ZIF-8/HA NPs	PDT	Overcomes the PDT induced hypoxia to further increase PDT efficiency	HeLa cell and SMMC7721 cell	[135]
	Bioinspired oxygen nanocarrier (C@HPOC)	PDT	Destroyed the primary tumors and effectively inhibited distant tumors and lung metastasis	4T1 tumor cells	[136]
	FA/PtBSA@MB-MSNS nanopolymers	PDT	Overcomes the PDT induced hypoxia to further increase PDT efficiency	4T1 tumor cells	[137]
PDA	PhMOSN@DOX-HA	PTT/CL	Biocompatible and biodegradable	4T1 tumor cell	[114]
	MoSe_2_@PDA nanocomposites	PTT/CL	High loading efficiency and pH-responsive Dox release effect	U14 cell	[115]
	PDA@DOX NPs	PTT/CL	Targeted and high drug load	4 T1 tumor cell	[116]
	PDA-Dox-Pc-QRH NPs	PDT/CT	Chemical photodynamic combined targeted therapy	A431 cell	[138]
	MnO_2_-PDA nanoshell	PDT/CT	Has the effect of alleviating tumor hypoxia and enhancing the PDT effect	B16F10 cell	[139]
RNA	CuS-RNP/DOX@PEI	PTT/GT/ CT	Provides a potential strategy to reduce tumor thermal tolerance for enhanced mild-PTT effects	A375 cell	[119]
	PPTC/siRNA	PDT	ROS-responsive siRNA release mode, PDT combined with RNAi to improve the therapeutic efficiency	HepG2 cells	[144]
DNA	DNA-UCNP-Au hydrogels	PTT	Low cytotoxicity and injectable properties allow local therapy to precisely target the tumor site	T24 bladder cancer cell	[121]
	UCNP-ApDz-TMPyP_4_ Nanoplatform	PTT	Combining PDT with gene therapy shows excellent antitumor effect	Catalysis	[141]
	DNA Nanosponge	PDT/GT	Effective against hypoxia-related photodynamic resistance during PDT in vivo with good biocompatibility	HeLa cells	[142]
Enzyme	GOIGLs (GOx)	PTT	Achieves milder PTT with good biocompatibility	A549 cell	[124]
	PMF−GOx	PTT/CDT	Fenton reaction and moderatehyperthermia cancer treatment together	MCF-7 tumor cell	[125]
	NC@GOx NPs	PTT/CDT	Combination therapy of starvation therapy, PTT and CDT for tumors	4T1 tumor cell	[126]
	MYR@HGNs (MYR)	PTT	Protect and activate the encapsulated enzymes for localized chemo-photothermal therapy	4T1 tumor cell	[127]
	HA-CAT@aCe6 (CAT))	PDT	Overcome tumor cell hypoxia during PDT and improve the therapeutic effect of PDT	MDA-MB-231 cell	[146]
	GOx-MSN@MnPc-LP	PDT	Good enzyme activity, and good therapeutic effect and safety through the synergistic treatment	4T1 tumor cell	[147]
	nanogel	PDT	Provide a new method for constructing combinatorial therapeutics with good tumor targeting ability and efficient anti-cancer effect	4T1 tumor cell	[148]

## Atomistic simulations of PTT and PDT nanomaterials

Simulation methods such as density-functional theory (DFT), molecular dynamics (MD), and Monte Carlo have been widely used to study the physical and chemical properties of nanomaterials. Ho7wever, the rational elucidation of biomimetic synthesis mechanisms is largely limited by the necessity to reach very long simulation times, while, at the same time, maintaining the capability of predicting the occurrence of chemical reactions. However, several studies by our own groups have dealt with the fundamental modelling of hybrid bio-inorganic interfaces by means of classical MD [149–151].

A few computational studies have also focused on photoactive biomimetic organic nanomaterials. Organic photothermal nano-agents, based on the electron-deficient thiadiazolobenzotriazole (TBZ) core and 2-(1,2,2-triphenylvinyl)thiophene, have been synthesized by Guo and co-workers, and their properties have been studied using the DFT and MD simulations. The simulations demonstrated that the conformation of the molecules, the band gap of materials, and the extinction coefficient influenced the light absorption [152]. In the case of polymers, Chen et al*.* showed that conjugated polymers facilitated the ROS generation compared to its monomers for PDT; the calculations were carried out using time-dependent density-functional theory (TDDFT) [153].

Qin and co-workers analyzed the Bader charge and electronic band structure of single iron atoms that anchored onto defective carbon dots confined in a biocompatible mesoporous silica nanoreactor by means of DFT calculations. They suggested that a high photothermal performance could be obtained due to an increased electron density and narrow bandgap of the iron atoms. Their calculations showed that there is a charge transfer between single Fe atoms and defected graphene compared to the pristine defected graphene [154].

A mathematical framework coupling a Monte-Carlo photo-transport model with Penne’s bioheat transfer model was used by Wang et al*.* for calculating the temperature increases of liquid metal NPs [155]. The heat generation of metallic NPs has been estimated by Beik et al*.,* who developed computational models to describe the plasmonic excitation of single AuNP and heat generation after laser illumination in a finite-element modelling framework, such as in COMSOL Multiphysics. They compared different sizes and shapes and suggested that the GNRs with 91 nm was optimal for heat generation [156].

Besides, the heat transfer of 3 nm AuNPs to a water pool was simulated with MD Simulation by Chen and co-workers [157]. Their results suggested that the optical energy was rapidly absorbed, and the temperature of AuNPs was raised, and then the energy was transmitted to the surrounding aqueous media through particle thermal expansion, particle melting, and vapor bubble formation. They used the large-scale atomic/molecular massively parallel simulator (LAMMPS) code, employing an embedded-atom potential for the metal particles and the TIP3P water model. Some remarks were that the water model, the interfacial wetting, and the boundary conditions affected the results significantly.

Jersey et al*.* developed a one-step approach for modeling the spatial thermal dose in a skin cancer model using Monte Carlo simulations and estimated the correlation between the concentration of nanomaterials in the tumor and the heat released in the tissue due to the photothermal process [158].

## Conclusions and perspectives

In this review, we summarized various methods for biomimetic synthesis of BBNMS. We introduced common BBNMs and their applications in PTT and PDT, mainly in the biomedical and cancer-treatment fields. The latest literature reports that biomolecules used in the synthesis of nanomaterials have several advantages, such as the tuning of NP morphology and reactivity, multiple interactions with their surrounding, sequence-dependent enhancement of electron and thermal transport, decreased toxicity, and biodegradability, while maintaining or improving their efficacy compared to the nanomaterials synthesized by other methods. Synergic effects are the most explored approach nowadays, where the nanomaterials are not only designed for reaction to the radiation stimulus but also to have different effects such as site-of-action targeted recognition, overcoming the hindrance of hypoxia, and synergistic chemotherapeutic drug delivery.

In future studies, the unique functions of biomolecules will be further developed. Target recognition, bio-catalysis, biocompatibility, and biodegradability could be the key to applying biomolecule-mimetic nanomaterials. We identified several key areas with a high potential for improving biomimetic synthesis for photothermal therapies.

First, the synthesis strategy of biomolecules as substrates for fabricating PTT and PDT nanomaterials by loading photothermal conversion materials requires further development. For example, the use of polypeptide nanomaterials as carriers of photothermal or photodynamic therapeutic agents remains to be studied, and there are few studies on stable 2D polypeptide nanomaterial carriers. The inherent characteristics of biomolecules determine the efficiency in vivo, so it is of great significance to develop various biomolecules to self-assemble to form different dimensional nanomaterials as carriers for therapeutic agents.

Second, a rationally designed therapeutic nanodrugs with stable function in the complex human environment could be developed, to avoid being decomposed by various metabolic and hydrolysis processes in the organism. To this end, the combination of experimental research with atomistic simulations, which have not been applied much to the study of PTT and PDT material, could be advantageous.

Third, the precise targeting ability of nanotherapeutic drugs in vivo and whether the PTT and PDT materials can stably act on the tumor sites needs to be answered.

Fourth, it is necessary to perform more studies on the strategies for improving the efficiency of PTT and PDT in cancer by enhancing the photothermal conversion ability and ROS generation.

Finally, we have suggested in the above presentation that the combination of PTT/PDT with other therapeutic methods could improve the effect of cancer treatment. Therefore, developing novel materials with high-performance photothermal and biological properties is useful via biomolecular design and controllable self-assembly strategies. On this basis, unique characteristics of biomolecules should be further developed to achieve economical, green, mild, and efficient combination therapy of diseases.

## Data Availability

Not applicable, refer to the original references.
